# Research on fault diagnosis of substation grounding grid based on graph neural network and multi source information fusion

**DOI:** 10.1371/journal.pone.0349513

**Published:** 2026-06-10

**Authors:** Xinghai Pu, Hao Ding, Lan Zhong, Xiaolin Chen

**Affiliations:** State Grid Jiangsu Electric Power Co., Ltd. Extra-high Voltage Branch Company, Nanjing, China; Aalto University, FINLAND

## Abstract

Hidden faults in substation grounding grids pose a serious threat to the safe and reliable operation of power systems. Traditional fault diagnosis methods ignore the correlation between grounding grid topology structures, resulting in low fault location accuracy. To address this issue, this study proposes a high-precision fault diagnosis and localization method that integrates graph neural networks (GNNs) with multi-source information. Innovatively abstracting the physical structure of the substation grounding grid into a topological graph model, with connection points and grounding electrodes as nodes, and conductor segments and their properties as edges, accurately representing the topological relationship of the grounding grid. A multimodal graph neural network (MM-GNN) was designed based on this, which captures topological features through graph convolution operations guided by grounding grids and adaptively integrates multi-source monitoring data such as electrical, infrared thermal imaging, and electromagnetic fields. In addition, this article innovatively developed an end-to-end conductor level positioning mechanism, incorporating physical connection rules into model training using topological constraint loss functions to ensure accurate output of fault locations. The experimental results based on a 220kV substation show that the proposed method significantly improves the diagnostic accuracy and provides an effective solution for the diagnosis and localization of grounding grid faults.

## 1. Introductions

The grounding grid, as a crucial facility in the power system [1,2], was responsible for key functions such as fault current discharge, equipment protection, and personal safety protection. Due to the long-term burial of the grounding grid underground, it was susceptible to hidden faults such as fracture, corrosion, and poor contact caused by factors such as soil corrosion, electrochemical reactions, mechanical stress, and temperature changes [3,4]. These faults develop slowly and are difficult to detect, usually difficult to detect under normal operating conditions. Once exposed under fault conditions, they may cause equipment damage, widespread power outages, and even threaten personal safety [5,6].

At present, numerous scholars have conducted extensive research on the diagnosis and localization of grounding grid faults [7–9]. Existing methods mainly included electrochemical diagnostic methods, electromagnetic field theory based diagnostic methods, signal injection, and machine learning technology paths. Electrochemical methods can only detect local areas and require high instrument costs for detection. The diagnostic method based on electromagnetic field theory used instruments to detect the magnetic induction intensity distribution of the entire grounding grid [7]. This method required high instrument accuracy, large detection workload, and complex electromagnetic environment interference measurement accuracy in substations. Injecting AC excitation may also cause relay protection mis-operation. This type of method still posed certain difficulties in achieving precise fault localization. Reference [8] proposed a fault diagnosis method based on square waves, which constructs a frequency model of the grounding system and injects different frequency square wave sources to analyze the output signals to achieve fault location. In recent years, machine learning technology has been widely applied in the field of grounding grid fault diagnosis. Literature [9] proposed a substation grounding grid model, which adopted advanced optimization algorithms to solve the problem on the basis of comprehensive consideration of the performance parameters of the grounding grid. Reference [10] proposed a magnetic field sensing method based on unmanned aerial vehicles for diagnosing grounding grid faults, significantly reducing operational risks and improving efficiency. However, the movement of drones introduces time-varying electromagnetic interference from substation equipment and the drones themselves, making the isolation of grounding network signals more complex. In addition, the combination of virtual sample generation technology and probabilistic neural network in reference [11] improved the fault recognition rate. Reference [12] used CDEGS software to establish a grounding grid fault analysis model, proposes an intelligent algorithm based on grey correlation degree and fuzzy theory, and applies this algorithm to the diagnosis and localization of grounding grid fault status. Researchers have proposed a solution using artificial neural network for grounding network detection [13], and studied the effectiveness of this method in rapid imaging of apparent resistivity through actual measurement of substation grounding network.

In recent years, abstracting the physical connections of grounding grids into graph structures has become the mainstream paradigm in this field, and the application of graph neural networks (GNNs) has been further developed on this basis. The core of this type of research lied in how to define the nodes, edges, and their characteristics of the graph to accurately reflect the topology and corrosion state of the grounding grid. Reference [14] proposed a Neighbor Selection and Merge Fault Diagnosis (NSMFD) strategy aimed at identifying fault nodes that capture graph structure information and node feature information. Compared to this, existing grounding system fault diagnosis methods heavily rely on theoretical models, resulting in unsatisfactory actual performance. Reference [15] established a theoretical model for the grounding system of cable circuits with shared grounding grids in various states, and combined it with attention mechanisms. However, most of these methods relied on the assumption of homogeneous graphs, that is, the types of nodes and edges are single, and fail to fully integrate multi-source and heterogeneous monitoring data in grounding grid diagnosis. Their diagnostic ability was largely limited by the representation ability of a single electrical feature.

In order to overcome the limitations of a single data source, another type of research has explored GNN models that integrate multiple sources of information, aiming to build more comprehensive and robust diagnostic systems. The challenge of such research lied in how to effectively integrate non topological heterogeneous data with graph models. Researchers have proposed an adaptive fault prediction and intelligent diagnosis method based on multi-level spatiotemporal graph neural networks to solve the difficulty of multi-source data fusion [16]. Reference [17] provided a comprehensive overview of GNNs in the power system, elaborating on several classic paradigms of GNN structures. A detailed review was conducted on key applications in the power system, including fault scenario applications, time series forecasting, power flow calculations, and data generation. Although these multi-source fusion methods have made progress, their model complexity is high and they are sensitive to noise and inconsistency issues between different source data. How to design lightweight and robust fusion mechanisms remains a challenge in current research.

Based on the above issues, this paper proposes a grounding grid fault diagnosis and localization method based on graph neural network (GNN) and multi-source information fusion. This method abstracts the physical structure of the grounding grid into a topological graph structure, where nodes represent connection points and grounding electrodes, and edges represent conductor segments and their properties, achieving effective mapping from physical structure to graph structure data. On this basis, a multimodal graph neural network architecture was designed, which adaptively aggregates neighborhood fault features through graph convolution operations guided by grounding grids, and dynamically weights and integrates multi-source heterogeneous information such as infrared thermography and electromagnetic fields using gating mechanisms. In addition, this article introduces a temporal dynamic fault feature learning mechanism to capture the evolution law of faults, and develops a conductor level end-to-end locator, using a topological constraint loss function to ensure the rationality and engineering practicality of the localization results.

This method has essential differences from existing methods in terms of topology modeling, graph convolution design, and multi-source fusion. In terms of topological modeling, this article models conductor segments as graph edges and introduces their physical properties (length, resistivity, cross-sectional area) as edge features, which differs from the existing GNN method that only considers conductor segments as node connections, directly providing data basis for conductor level localization. In the design of graph convolution, this article uses resistance distance weighting and potential gradient driving mechanisms to make the neighborhood aggregation weights conform to Ohm’s law, which compensates for the shortcomings of standard GAT and other methods that rely entirely on data-driven approaches and lack physical interpretability. There are two limitations to existing methods in multi-source fusion: ①Attention based fusion (such as Transformer cross modal attention) calculates global weights in the feature dimension and cannot distinguish modal quality differences between different samples within the same batch. ② Although gated fusion methods (such as GRU gating) can dynamically modulate features, gated signals were generated from single modal features and lack explicit modeling of cross modal correlations. The sample adaptive fusion mechanism proposed in this article learns the modal correlation vector independently for each sample, so that the fusion weight directly reflects the actual quality and complementarity of each modal signal in that sample, and has stronger sample level robustness in scenarios with uneven sensor noise or partial modal degradation. In addition, the end-to-end localization mechanism embeds physical connectivity constraints into the optimization objective through a topological constraint loss function, avoiding the error accumulation of traditional two-stage methods.

## 2. Topology modeling and data processing of grounding network

### 2.1. Construction of topology map

This paper abstracts the physical structure of the grounding grid as a mathematical graph model, and uses graph theory representation methods to effectively map the complex geometric shape of the grounding grid to structured data. In the graph theory framework, the grounding grid can be represented as an undirected graph *G*, as follows:


$$\begin{array}{c} G=\left(V,\varepsilon \right) \end{array}$$ (1)

Where *V* represents the set of nodes, and *ε* represents the set of edges.

**Fig 1:**
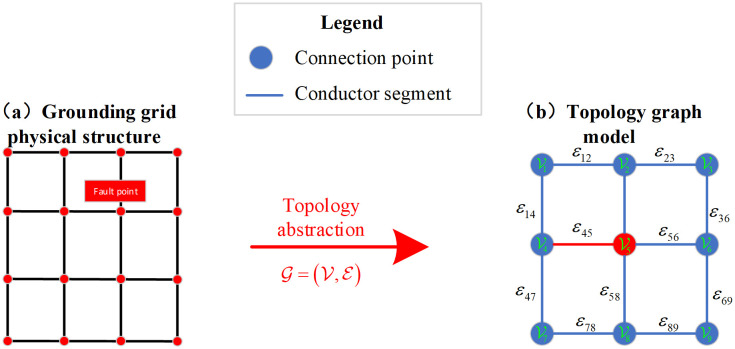
Schematic diagram of the conversion from the physical structure of the grounding grid to topology diagram.

This article abstracts the grounding grid as an undirected graph, which has sufficient physical rationality in the scenario of power frequency fault diagnosis. Firstly, the fault diagnosis adopts steady-state power frequency measurement (50/60 Hz), and the fault current is discharged through multiple parallel paths. The key information required for diagnosis is the spatial distribution pattern of the current amplitude rather than the instantaneous direction. The equivalent conduction relationship under power frequency steady state is essentially bidirectional symmetric. Secondly, the mutual inductance coupling between conductors mainly affects transient high-frequency processes. Under power frequency conditions, the assumption of quasi-static field holds: at50Hz, the electromagnetic wave wavelength *λ* = *c*/*f* = 3 × 10⁸/50 ≈ 6000 km. However, the coverage area of the substation grounding network studied in this paper is about 120-180m, and the ratio of size to wavelength is about 3 × 10 ^−5^(≪1), which satisfies the quasi-static approximation condition and is consistent with the common processing method for low-frequency electromagnetic field analysis in power systems. Therefore, the main coupling between conductors is achieved through the resistive coupling of soil, which is reflected in the graph model through node potential and edge resistance properties. The conversion process from the physical structure of the grounding grid to the topology diagram is shown in Fig 1. In this mapping process, nodes represent the conductor connection points and grounding electrodes in the grounding grid, while edges represent the conductor segments connecting adjacent nodes. Therefore, the vector or matrix dimensions related to the conductor segments are represented by |ϵ|.

### 2.2. Data feature system

Considering the diversity and complexity of the manifestations of grounding grid faults, a single type of monitoring data often cannot fully reflect the true state of the fault [18,19]. This article constructs three complementary monitoring systems: electrical parameter measurement, infrared thermal imaging detection, and electromagnetic field distribution measurement, which capture the operational status information of the grounding grid from different dimensions. The characteristic parameters and fault sensitivity of the data source are shown in Table 1.

**Table 1:** Multi-source data feature system.

Data type	Main characteristic parameters	Fault susceptibility	Physical mechanisms
Electrical parameters	Grounding resistance, Node potential, Branch current	High	Conductor corrosion and breakage directly affect electrical characteristics
Infrared thermal image	Temperature distribution, Hot spot location, Temperature gradient	Medium	Fault point resistance increase causes local overheating
Electromagnetic field distribution	Magnetic flux density, Magnetic field vector, Spatial gradient	Medium	Current path change causes abnormal magnetic field distribution

Through collaborative monitoring of multi-source data, the existence and location of faults can be verified from different physical dimensions, which is beneficial for improving the reliability and accuracy of diagnostic results.

### 2.3. Multimodal feature extraction and fusion

#### 2.3.1. Feature engineering method.

Electrical parameter feature engineering includes time-domain statistical features and frequency-domain feature. Let the continuous time-domain signal be *x*(*t*), and obtain a discrete signal sequence $\overset{N}{\underset{i=\text{1}}{\left\{x_{i}\right\}}}$ through sampling. Where *x*_*i*_ = *x*(*t*_*i*_) represents the signal value *t*_*i*_ at the *i*-th sampling time, and *N* is the number of sampling points. Time domain statistical features include mean features and variance features. The mean feature is represented as:


$$\begin{array}{c} \mu =\frac{\text{1}}{N}\sum_{i=1}^{N}x_{i} \end{array}$$ (2)

Where *μ* is the signal mean, reflecting the degree of signal dispersion.

The variance features are represented as follows:


$$\begin{array}{c} \sigma^{\text{2}}=\frac{\text{1}}{N}\sum_{i=1}^{N}(x_{i}-\mu {)}^{\text{2}} \end{array}$$ (3)

Where *σ*^2^ is the signal variance.

The power spectral density is expressed as:


$$\begin{array}{c} P\left(k\right)=\frac{{\left|X\left(k\right)\right|}^{\text{2}}}{N} \end{array}$$ (4)

Where *P*(*k*) represents the power spectral density of the *k-*th frequency component, and X(k) represents the complex value of the time-domain signal *x*(*n*) at the *k*-th frequency point after fast Fourier transform.

The infrared thermal imaging feature adopts a gray level co-occurrence matrix, and its contrast texture feature is represented as:


$$\begin{array}{c} C=\sum_{i,j}|i-j{|}^{\text{2}}P\left(i,j\right) \end{array}$$ (5)

Where *C* is the contrast texture feature value, *i* and *j* are the grayscale values of the pixel pair, *P*(*i*, *j*) is the normalized grayscale co-occurrence matrix element, $|i-j{|}^{\text{2}}$ is the square of the grayscale difference.

Extract magnetic field spatial gradient features, represented as:


$$\begin{array}{c} \nabla \text{B=}\left(\frac{\partial \text{B}}{\partial x},\frac{\partial \text{B}}{\partial y},\frac{\partial \text{B}}{\partial z}\right) \end{array}$$ (6)

Where ∇B is the gradient of the magnetic induction intensity vector *B*, and $\frac{\partial B}{\partial x}$, $\frac{\partial B}{\partial y}$, and $\frac{\partial B}{\partial z}$ are the partial derivatives of the magnetic induction intensity in the *x*, *y*, and *z* coordinate directions, respectively. To address the issue of dimensional differences and inconsistencies between multimodal features, mutual information based feature selection methods [20] and principal component analysis dimensionality reduction techniques are employed [21].

#### 2.3.2. Data alignment and standardization.

Multimodal data fusion requires standardized alignment of time, space, and numerical scales. Time alignment is based on high-frequency sampling of electrical parameters and compensates for missing data through cubic spline interpolation, expressed as:


$$\begin{array}{c} x\left(t\right)=a_{i}(t-t_{ci}{)}^{\text{3}}+b_{i}(t-t_{ci}{)}^{\text{2}}+c_{i}(t-t_{ci})+d_{i} \end{array}$$ (7)

Where *x*(*t*) is the interpolation result at time *t*, *t*_*ci*_ is the time of the *i*-th known data point, *a*_*i*_, *b*_*i*_, *c*_*i*_ and *d*_*i*_ are the coefficients of the *i*-th third-order polynomial.

The Z-score standardization method is used to normalize the zero mean and unit variance of each modal feature vector, which is expressed as:


$$\begin{array}{c} x_{norm}=\frac{x-\mu_{f}}{\sigma_{f}} \end{array}$$ (8)

Where *μ*_*f*_ and *σ*_*f*_ are the mean and standard deviation of the features, respectively.

## 3. Multimodal graph network neural design

### 3.1. Overall architecture design

This paper designs a Multi-modal Graph Neural Network (MM-GNN) architecture. This network is based on the topology diagram of the grounding grid and introduces a multimodal perception mechanism. It integrates multiple features such as electrical parameters, infrared thermal imaging, and electromagnetic field data to achieve high-precision identification of grounding grid faults and precise positioning of conductor levels.

**Fig 2:**
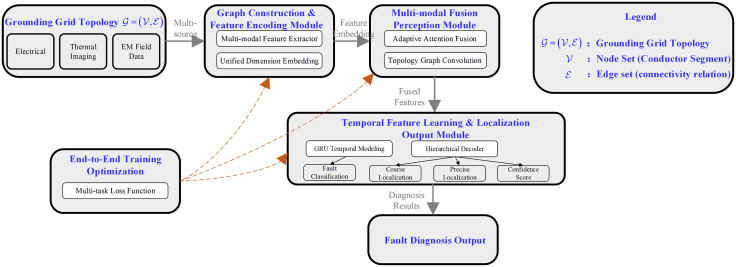
Schematic diagram of the conversion from physical structure of the grounding grid to topology diagram.

The overall architecture of MM-GNN adopts an end-to-end design concept, consisting of three modules in series: graph construction and feature encoding, multimodal fusion perception, temporal feature learning, and localization output. Joint optimization is carried out through a multi task loss function. Fig 2 shows the overall architecture of the network.

The graph construction and feature encoding module serves as the input of the network [22,23] responsible for converting multimodal raw data into tensor representations that can be processed by the network; The multimodal fusion perception module is located in the middle layer of the network, which achieves deep fusion of multi-source heterogeneous data and generates a unified feature representation; The temporal feature learning and localization output module extracts the temporal evolution law of faults and outputs diagnostic and localization results.

### 3.2. Topology guided graph convolution

Based on Section 2.1, the grounding network diagram *G*=(*V*, *ε*) is established, where the node set *V* represents the connection point and grounding electrode, and the edge set *ε* corresponds to the conductor segment. Each edge *e*_*ij*_ ∈ *ε* is accompanied by an attribute vector ***a***_*ij*_, which includes physical quantities such as conductor length, material, and resistance value. For node *v*_*i*_, its feature update process can be represented as:


$$\begin{array}{c} \overset{\left(l+1\right)}{\underset{i}{\text{h}}}=\sigma \left(\sum_{j\in 𝒩\left(i\right)}\overset{\left(l\right)}{\underset{ij}{\alpha }}\overset{\left(l\right)}{\underset{\text{topo}}{\text{W}}}\overset{\left(l\right)}{\underset{j}{\text{h}}}+\overset{\left(l\right)}{\underset{\text{self}}{\text{W}}}\overset{\left(l\right)}{\underset{i}{\text{h}}}\right) \end{array}$$ (9)

Where $\overset{\left(l\right)}{\underset{i}{h}}$ represents the eigenvector of node *v*_*i*_ at layer *l*, *N*(*i*) is the set of neighboring nodes of node *i*, $\overset{\left(l\right)}{\underset{ij}{\alpha }}$ is the topology aware attention weight, $\overset{\left(l\right)}{\underset{topo}{W}}$ and $\overset{\left(l\right)}{\underset{self}{W}}$ are the neighbor aggregation weight matrix and self update weight matrix of layer *l*, respectively, and σ(•) is the activation function.

This section explicitly introduces the conductor length *L*_*ij*_, resistivity *ρ*_*ij*_, and inter node potential difference Δ*V*_*ij*_ into attention weight calculation. *L*_*ij*_ and *ρ*_*ij*_ are extracted from the edge attribute vector *e*_*ij*_, while $\Delta V_{ij}=\left|V_{i}-V_{j}\right|$ is calculated from the potential components in the node feature vectors $\overset{\left(l\right)}{\underset{i}{h}}$ and $\overset{\left(l\right)}{\underset{j}{h}}$. In the proposed TGGC attention mechanism, the inter node potential difference Δ*V*_*ij*_ represents the potential difference between adjacent nodes *i* and *j* in the grounding grid, and is an electrical quantity with clear physical meaning. In actual grounding grid detection and evaluation, node potential can usually be obtained by injecting test current into the grounding grid and measuring the potential value relative to the remote reference point at each node position, thereby calculating the potential difference between nodes. In this study, the node potential values were obtained through electromagnetic field simulation. Specifically, a three-dimensional electromagnetic field model of the grounding grid is established based on ANSYS Maxwell. The potential distribution of the grounding grid is calculated under given excitation and fault conditions, and the potential values of each node are extracted. The above-mentioned node potential, as one of the key electrical parameters, together with information such as grounding resistance and branch current, forms a node feature vector, which is further fused with infrared thermal imaging features and electromagnetic field features for the calculation of TGGC attention weights. Topology aware attention weights $\overset{\left(l\right)}{\underset{ij}{\alpha }}$ integrate physical attributes and learning feature representations as follows:


$$\begin{array}{c} \overset{\left(l\right)}{\underset{ij}{\alpha }}=\frac{exp\left(\alpha \overset{\left(l\right)}{\underset{ij}{f}}-\beta \frac{L_{ij}\rho_{ij}}{\left|\Delta V_{ij}\right|+\varepsilon }\right)}{\sum_{k\in 𝒩\left(i\right)}exp\left(\alpha \overset{\left(l\right)}{\underset{ik}{f}}-\beta \frac{L_{ik}\rho_{ik}}{\left|\Delta V_{ik}\right|+\varepsilon }\right)} \end{array}$$ (10)

Where $\overset{\left(l\right)}{\underset{ij}{f}}={\left(w^{\left(l\right)}\right)}^{T}\left[\overset{\left(l\right)}{\underset{i}{h}}\|\overset{\left(l\right)}{\underset{j}{h}}\right]$ represents the learned correlation of base point features, with *α* = 1 and *β* = 0.5 as balancingcoefficients, and $\varepsilon =\text{1}\times {\text{1}\text{0}}^{-\text{6}}$ as a numerical stability constant. The physical significance of this formula lies in the quantitative influence of resistance. The terms *L*_*ij*_*ρ*_*ij*_ in the numerator denote the resistance contribution of the conductor segment. The physical relationship for the total resistance of the conductor is:


$$\begin{array}{c} R_{ij}=\frac{\rho_{ij}L_{ij}}{A_{ij}} \end{array}$$ (11)

Where *R*_*ij*_ represents the resistance of the conductor segment between nodes *i* and *j*, *A*_*ij*_ represents the cross-sectional area of the conductor.

When the cross-sectional areas *A*_*ij*_ of each conductor segment are approximately equal, it can be approximated as *R*_*ij*_ = *ρ*_*ij*_*L*_*ij*_. Since the coefficient of this term in the attention weight formula is negative, the higher the resistance, the lower the corresponding weight. The balancing coefficients *α* = 1 and *β* = 0.5 were determined by grid-search on the validation set, as detailed in Section 4.1. Through this design, the conductor length, resistivity, and potential difference quantitatively adjust the feature aggregation weight, ensuring that the graph convolution operation aligns with the physical laws of current distribution in the grounding grid.

The design rationale of Equation (10) differs fundamentally from standard graph attention networks such as GAT, which compute attention weights solely from learned node feature similarities and contain no physical constraints. Equation (10) instead encodes two domain-specific physical priors of grounding grids. First, by Ohm’s law, higher conductor resistance impedes current flow; corroded conductors with elevated resistivity therefore carry less current and should receive lower aggregation weights, reflected by the negative coefficient of the $L_{ij}\rho_{ij}$ term. Second, the potential gradient is the driving force of current; a path with a larger inter-node potential difference $\left|{\Delta V}_{ij}\right|$ carries greater current and should exert stronger influence on neighbourhood aggregation, achieved by placing $\left|{\Delta V}_{ij}\right|$ in the denominator so that larger potential differences reduce the suppressive effect of the resistance term and raise the weight. These two priors cannot be reliably inferred from labelled data alone given the limited fault samples available in practical substation settings; their explicit incorporation into the attention formula constitutes a principled physics-guided inductive bias that improves both physical interpretability and data efficiency. The selection of the balancing coefficients alpha and beta, as well as the time-window length T and LSTM architecture, is detailed in Section 4.1 through systematic grid-search ablation on the validation set.

A multi-scale aggregation mechanism is introduced to consider neighbor information with different hop counts and capture remote dependencies in the grounding grid, represented as:


$$\begin{array}{c} \overset{\left(l+1\right)}{\underset{i}{\text{h}}}=\text{COMBINE}\left(\{{\text{AGGREGATE}}_{k}\left(\{\overset{\left(l\right)}{\underset{j}{\text{h}}}:j\in {𝒩}_{k}(i)\}\right):k=1,2,\ldots ,K\}\right) \end{array}$$ (12)

Where *N*_*k*_(*i*) represents the *k*-hop neighbor set of node *i*, where *K* represents the maximum number of hops. AGGREGATE_*k*_(•) and COMBINE(•) are the *k*-hop aggregation function and combination function, respectively.

### 3.3. Adaptive multimodal fusion

The multimodal fusion network proposed in this article adopts a hierarchical architecture design and encodes features for each modality. For the electrical parameter mode X_elec_, a fully connected network is used. For the thermal infrared image modality X_ther_, a convolutional neural network is used. For the electromagnetic field distribution X_emag_, a 3D convolutional network is used to obtain feature representations F_elec_, F_ther_ and F_emag_, respectively.

After obtaining the features of each modality, the adaptive fusion module calculates the importance weight of each modality through attention mechanism. A modal correlation vector *R*_*m*_ is introduced. *R*_*m*_ is used to provide auxiliary information for adaptive weight calculation. It should be noted that *R*_*m*_ is not a pre-set fixed parameter, but is dynamically generated from the feature representations of various modalities *F*_*m*_ through a learnable mapping function *f*_*m*_(·) based on samples (i.e. *R*_*m*_ = *f*_*m*_(*F*_*m*_)), and its parameters are optimized through backpropagation during training. Considering the correlation between modalities and the quality of each modality feature, the adaptive weight calculation is as follows:


$$\begin{array}{c} w_{m}=\frac{exp\left(MLP_{attn}\left([F_{m};\text{mean}(R_{m});\text{std}(F_{m})]\right)\right)}{\sum_{m}exp\left(MLP_{attn}\left([F_{m};\text{mean}(R_{m});\text{std}(F_{m})]\right)\right)} \end{array}$$ (13)

Where *w*_*m*_ represents the adaptive weight of mode *m*, *m*∈ {elec, ther, emag} represents the electrical parameter mode, thermal infrared mode, and electromagnetic field mode, *F*_*m*_ represents the feature representation of mode *m*, *R*_*m*_ represents the correlation vector between mode *m* and other modes, mean(*R*_*m*_) and std(*F*_*m*_) represent the mean of the correlation vector and the standard deviation of the feature vector, respectively, *MLP*_*attn*_(•) represents a multi-layer perceptron for attention calculation.

The final fusion features are obtained through weighted combination and cross connection:


$$\begin{array}{c} F_{fused}=\sum_{m}w_{m}F_{m}+MLP_{cross}\left(\left[F_{\text{elec}};F_{\text{ther}};F_{\text{emag}}\right]\right) \end{array}$$ (14)

Where *F*_*fused*_ represents the final fused feature representation, and *MLP*_cross_(•) represents a cross connected network that captures nonlinear interaction information between modalities.

### 3.4. Spatiotemporal feature learning

To capture this dynamic evolution process, this paper designs a temporal dynamic fault feature learning mechanism, which combines long short-term memory networks (LSTM) and graph convolutional networks (GCN) to achieve joint learning of spatiotemporal features. To improve the interpretability and reproducibility of the method, the following provides a detailed explanation of the input structure, time window partitioning criteria, and specific computational logic for spatiotemporal feature interaction of LSTM.

(1) Time series data organization and time window design. The monitoring data of the grounding grid is organized in chronological order into a graph sequence {*G*_1_, *G*_2_, …, *G*_T_}, where the graph *G*_T_ =(*V*, *E*, *X*_*t*_) at each moment has the same topological structure (node set *V* and edge set *E* remain unchanged), but the node feature matrix *X*_*t*_ varies over time. Here *X*_*t*_ ∈ *R*^*N*×*d*^ represents the feature matrix of all *N* nodes at time *t*, where *d* is the feature dimension of each feature node.

The selection of time window length *T* is determined based on a systematic analysis of fault evolution characteristics from the simulated fault dataset. Specifically, for the 8,000 single-fault samples generated by the ANSYS Maxwell simulation platform, we extracted the time series of grounding resistance change rate (*dR*/*dt*) and node potential anomaly index for each fault type. Statistical analysis revealed that progressive corrosion faults exhibit a statistically significant resistance increase (exceeding 3-sigma threshold) within 24–72 hours, while intermittent faults such as loose connections show characteristic fluctuation periods of 1–6 hours. To cover the full observable evolution cycle of the slowest progressive fault while maintaining computational feasibility, a window of *T* = 48 (with a sampling interval of 1 hour), covering 2 days of continuous monitoring data, which can capture the slow changes of progressive faults and identify the rapid evolution of sudden faults.

(2) LSTM network structure and input dimension definition. For each node *v*_*i*_ in the grounding grid, its timing input is$X_{i}=\left[\overset{\left(\text{1}\right)}{\underset{i}{x}},\overset{\left(\text{2}\right)}{\underset{i}{x}},\cdots ,\overset{\left(T\right)}{\underset{i}{x}}\right]$. Where $\overset{\left(t\right)}{\underset{i}{x}}\in R^{d}$ represents the *d*-dimensional eigenvector of node *i* at time *t*, therefore $X_{i}\in R^{T\times d}$is the eigenvector of the node throughout the entire time window. The node feature dimension *d* is determined by the output of the multimodal fusion layer mentioned above. After the multimodal information adaptive fusion in section 3.2.2, the fusion feature dimension of each node is *d* = 64, which comprehensively includes the fusion representation of electrical parameters (grounding resistance, node potential, branch current), infrared thermal imaging features (temperature distribution, hotspot location), and electromagnetic field features (magnetic induction intensity, spatial gradient). The two-layer LSTM architecture with hidden dimensions of 128 and 64 was selected based on a grid-search ablation on the validation set. Single-layer configurations with hidden dimensions of 64 or 128 yielded validation F1 scores of 92.1% and 93.4%, respectively, whereas the two-layer (128 → 64) configuration achieved 95.8%, indicating sufficient capacity to capture both short-term fluctuations and long-term drift. Deeper architectures (three layers) showed no further improvement (+0.2%) but increased inference time by 38%. The progressive dimension reduction (128 → 64) follows the encoder-style bottleneck design commonly adopted in fault diagnosis sequence models, which compresses redundant temporal features while preserving discriminative fault patterns. This article uses a dual layer LSTM structure to encode temporal features: the first layer LSTM: input dimension *d* = 64, hidden layer dimension $h_{\text{1}}=\text{1}\text{2}\text{8}$, output $\overset{\left(\text{1}\right)}{\underset{i}{H}}\in R^{T\times \text{1}\text{2}\text{8}}$; the second layer LSTM: input dimension *d* = 128, hidden layer dimension $h_{\text{2}}=\text{6}\text{4}$, output $\overset{\left(\text{2}\right)}{\underset{i}{H}}\in R^{T\times \text{6}\text{4}}$. The LSTM unit follows a standard gating mechanism during the update process at each time step *t*:


$$\begin{array}{c} \begin{array}{c} \text{forget gate:}f_{t}=\sigma (W_{f}[h_{t-1},x_{t}]+b_{f})\\ \text{input gate:}i_{t}=\sigma (W_{i}[h_{t-1},x_{t}]+b_{i})\\ \text{candidate memory:}{\tilde{C}}_{t}=tanh(W_{c}[h_{t-1},x_{t}]+b_{c})\\ \text{memory update:}C_{t}=f_{t}⊙C_{t-1}+i_{t}⊙{\tilde{C}}_{t}\\ \text{input gate:}o_{t}=\sigma (W_{o}[h_{t-1},x_{t}]+b_{o})\\ \text{hidden state:}h_{t}=o_{t}⊙tanh\left(C_{t}\right) \end{array} \end{array}$$ (15)

Where σ represents the sigmoid activation function, ⊙ represents element wise multiplication, *W* and *b* are the learnable weight matrix and bias vector, respectively, and $\left[h_{t-\text{1}},x_{t}\right]$ represents the concatenation of the hidden state from the previous moment and the current input. Through this recursive structure, LSTM can model long-term dependencies in the time dimension and capture the evolutionary patterns of fault features.

(3) The computational logic of spatiotemporal feature interaction. In order to achieve effective fusion of spatial topological features and temporal evolution features, this paper designs a three-stage spatiotemporal interaction mechanism of “spatial aggregation temporal encoding feature fusion”.

Phase 1. Aggregation of spatial features. At each time *t*, the graph convolutional network is first used to aggregate neighborhood features on the graph $G_{t}$ at the current time and extract node spatial topological features. The spatial aggregation feature calculation for node *i* at time *t* is as follows:


$$\begin{array}{c} \overset{spatial\left(t\right)}{\underset{i}{h}}=\sigma \;\left(\sum_{j\in N\left(i\right)}\overset{\left(t\right)}{\underset{ij}{\alpha }}\text{\textbackslash{}hspace\{0.17em}\}W^{spatial}\text{\textbackslash{}hspace\{0.17em}\}\overset{\left(t\right)}{\underset{j}{x}}\right) \end{array}$$ (16)

Where N(i) represents the set of neighboring nodes of node *i*, $\overset{\left(t\right)}{\underset{ij}{\alpha }}$ represents the topological aware attention weight at time t (calculated by formula (10)), and $W^{spatial}\in R^{\text{6}\text{4}\times \text{6}\text{4}}$ represents the weight matrix of spatial aggregation. This step generates a spatial feature vector $\overset{\text{spatial}(t)}{\underset{i}{h}}\in R^{\text{6}\text{4}}$ that integrates neighborhood information for each node.

Phase 2. Temporal Feature Encoding. Input the spatial feature sequence of node i across all T time steps into theLSTM network to extract temporal dependency patterns: $\overset{temporal}{\underset{i}{h}}=\text{LSTM}\left(\left[{\overset{spatial}{\underset{i}{h}}}^{\left(\text{1}\right)},{\overset{spatial}{\underset{i}{h}}}^{\left(\text{2}\right)},\ldots ,{\overset{spatial}{\underset{i}{h}}}^{\left(T\right)}\right]\right)$, afterprocessing the entire sequence, the final hidden state $\overset{temporal}{\underset{i}{h}}\in R^{\text{64}}$ of the LSTM network serves as the temporal encoding feature for that node. This feature encapsulates the complete evolutionary information from time step 1 to time step T.

Phase 3. Spatio-Temporal Feature Fusion. The spatial features at the current time step are concatenated with the temporal encoding features. This concatenated feature vector undergoes a nonlinear transformation via a Multi-Layer Perceptron (MLP):


$$\begin{array}{c} \overset{final}{\underset{i}{h}}=\text{MLP}\left(\left[\overset{spatial\left(T\right)}{\underset{i}{h}}\;\|\;\overset{temporal}{\underset{i}{h}}\right]\right) \end{array}$$ (17)

Where || denotes the concatenation operation (resulting in a 128-dimensional output), MLP represents a two-layer fully connected network with the following structure: 128-dimensional input → 64-dimensional hidden layer (ReLU activation) → 32-dimensional output. The final output $\overset{final}{\underset{i}{h}}\in R^{\text{3}\text{2}}$ represents the spatiotemporal fusion feature representation for node i. The complete spatiotemporal feature interaction process can be described as follows:


$$\begin{array}{c} \left\{ \begin{array}{c} @l\text{Step }\text{1}:\overset{\text{spatial }}{\underset{i}{h}}(t)=\text{GCN}\left(X_{t},A\right),\\ \;\;\text{ Step }\text{2}:\overset{\text{temporal }}{\underset{i}{h}}=\text{LSTM}\left(\overset{\text{spatial }}{\underset{i}{h}}(\text{1}:T)\right),\\ \;\;\text{ Step }\text{3}:\overset{\text{final }}{\underset{i}{h}}=\text{MLP}\left(\left[\overset{\text{spatial }}{\underset{i}{h}}(T)∥\overset{\text{temporal }}{\underset{i}{h}}\right]\right). \end{array}\right. \end{array}$$ (18)

Where A represents the adjacency matrix of the grounding grid.

The output is the spatiotemporal fusion feature $\{\overset{final}{\underset{\text{1}}{h}},\overset{final}{\underset{\text{2}}{h}},\;\cdots ,\overset{final}{\underset{N}{h}}\;\;\}$ for all nodes.

(4) Multi-scale temporal modeling. This paper introduces a multi-scale time series modeling strategy employing three parallel branches to process time windows of varying lengths: Short-term branch: $T_{s}=\text{1}\text{2}$ hours, capturing rapid-change features, LSTM hidden dimension 32; Medium-term branch: $T_{m}=\text{4}\text{8}$ hours, capturing regular evolutionary features, LSTM hidden dimension 64; Long-term branch: $T_{l}=\text{1}\text{6}\text{8}$ hours (7 days), capturing slow trends, LSTM hidden dimension 32. The three branches independently process data within their respective time windows, outputting spatiotemporal fusion features: $h_{s}$, $h_{m}$, $h_{l}$. Subsequently, adaptive fusion is performed via a gated attention mechanism:


$$\begin{array}{c} \begin{array}{c} \left[\beta_{s},\text{\textbackslash{}hspace\{0.17em}\}\beta_{m},\text{\textbackslash{}hspace\{0.17em}\}\beta_{l}\right]=\text{Softmax}\left({\text{MLP}}_{\text{gate}}\;\left(\left[\text{\textbackslash{}hspace\{0.17em}\}h_{s}\;\|\;h_{m}\;\|\;h_{l}\text{\textbackslash{}hspace\{0.17em}\}\right]\right)\right)\\ h_{\text{multi-scale}}=\beta_{s}\text{\textbackslash{}hspace\{0.17em}\}h_{s}+\beta_{m}\text{\textbackslash{}hspace\{0.17em}\}h_{m}+\beta_{l}\text{\textbackslash{}hspace\{0.17em}\}h_{l} \end{array} \end{array}$$ (19)

Where $MLP_{gate}$ represents the gated network (input dimension 128, output dimension 3), $\beta_{s},\beta_{m},\beta_{l}$ denotes the adaptive weights for the three branches, satisfying $\beta_{s}+\beta_{m}+\beta_{l}=\text{1}$. This mechanism enables the model to automatically adjust its focus on features across different time scales based on specific fault types: for sudden faults (e.g., loose connections), the short-term branch weights are larger; for gradual faults (e.g., corrosion), the long-term branch weights are larger. *h*_*multi-scale*_ represents the fused multi-scale temporal feature vector. Formulas (17) – (19) describe the single branch spatiotemporal feature extraction process based on the main window *T* = 48, which outputs node level spatiotemporal fusion features $\overset{final}{\underset{i}{h}}\in R^{\text{3}\text{2}}$. On this basis, the multi-scale mechanism runs two auxiliary branches in parallel, short-term (*T*_*s*_ = 12) and long-term (*T*_l_ = 168). Each branch independently executes the same three-stage process of “spatial aggregation→temporal encoding→feature fusion” as formulas (17) – (19), generating corresponding spatiotemporal features *h*_*s*_ and *h*_*l*_, respectively. The outputs of the three branches are then fused by the gate controlled attention mechanism in formula (19) with adaptive weights *β*_*s*_, *β*_*m*_ and *β*_*l*_ to obtain the final multi-scale temporal feature *h*_*multi*_. This feature replaces the single branch output $\overset{final}{\underset{i}{h}}$ as the input for subsequent fault diagnosis and localization modules. This design ensures that the multi-scale mechanism expands the model’s ability to capture different fault evolution speeds through parallel branches without changing the main spatiotemporal feature extraction logic.

### 3.5. End to end positioning mechanism

#### 3.5.1. Topological constraint loss function.

The topological constraint loss function consists of three main components: basic localization loss, topological connectivity constraint loss, and spatial distance constraint loss. The basic positioning loss adopts the form of mean square error to measure the deviation between the predicted position and the actual fault position:


$$\begin{array}{c} L_{base}=\frac{\text{1}}{N}\sum_{i=1}^{N}∥\overset{pred}{\underset{i}{p}}-\overset{true}{\underset{i}{p}}\overset{\text{2}}{\underset{\text{2}}{∥}} \end{array}$$ (20)

Where $\overset{pred}{\underset{i}{P}}$ and $\overset{true}{\underset{i}{P}}$ represent the predicted fault location and true fault location of the *i*-th sample, respectively, and *N* is the total number of samples.

This constraint is achieved by calculating the distance from the predicted position to the nearest conductor segment, which is represented as:


$$\begin{array}{c} L_{topo}=\frac{\text{1}}{N}\sum_{i=1}^{N}{min}_{e\in E}d(\overset{pred}{\underset{i}{p}},e)\cdot 𝕀(d(\overset{pred}{\underset{i}{p}},e)>\tau ) \end{array}$$ (21)

Where *E* represents the set of all conductor segments in the grounding grid, *d*($\overset{pred}{\underset{i}{P}}$, *e*) represents the shortest distance from the predicted position $\overset{pred}{\underset{i}{P}}$ to the conductor segment *e*, *τ* is the preset distance threshold, and Ⅱ(•) is the indicator function.

The spatial distance constraint loss considers the physical distance relationship between adjacent nodes in the grounding grid to prevent unreasonable jumping phenomena in the positioning results. Taking into account the above three constraints, the final topological constraint loss function is defined as:


$$\begin{array}{c} L_{spatial}=\frac{\text{1}}{N}\sum_{i=1}^{N}\sum_{\begin{array}{c} e^{\prime }\in E\\ e^{\prime }\sim \;e_{i} \end{array}}\overset{\text{2}}{\underset{\text{2}}{\|\overset{pred}{\underset{i}{P}}-\overset{pred}{\underset{e^{\prime }}{P}}\|}} \end{array}$$ (22)

Where *e’* ~ *e*_*i*_ represents the direct connection between conductor segment *e’* and conductor segment *e*_*i*_ in the topology of the grounding grid, and $\overset{pred}{\underset{e^{\prime }}{P}}$ represents the predicted fault location coordinates mapped to adjacent conductor segment *e.’* This mapping is determined based on the geometric parameters of the conductor segments. Taking into account the above three constraints, the final topological constraint loss function is defined as:


$$\begin{array}{c} L_{total}=L_{base}+\lambda_{\text{1}}L_{topo}+\lambda_{\text{2}}L_{spatial} \end{array}$$ (23)

Where *λ*_1_ and *λ*_2_ are hyperparameters that balance the importance of different loss terms and are optimized using grid search methods.

**Fig 3:**
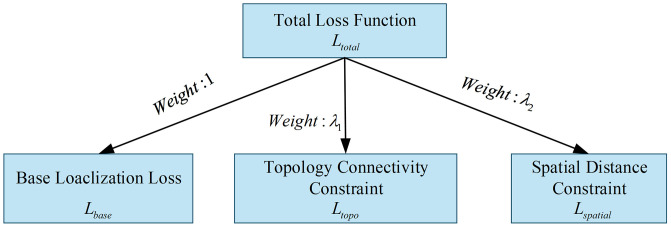
Topology constraint loss function components diagram.

Fig 3 shows the composition of the topological constraint loss function. This function consists of three core parts: the basic positioning loss *L*_*base*_ is used to measure the error between the predicted and true positions, the topological constraint loss *L*_*topo*_ restricts the predicted results to be located on the conductor path, and the spatial distance loss *L*_*spatial*_ restricts the physical jumps between adjacent nodes. Each item is dynamically adjusted through weights *λ*_1_ and *λ*_2_ to achieve a balance between physical rationality and positioning accuracy.

#### 3.5.2. Conductor level positioning output.

The conductor level positioning output adopts a two-stage positioning strategy. The first stage is conductor segment identification, which evaluates the fault probability of each conductor segment in the grounding network through a multimodal graph neural network. The network outputs a probability vector with a dimension of |ε|, where |ε| represents the total number of conductor segments in the grounding grid. The network outputs a probability vector *P*_*sement*_∈$R^{\left|\varepsilon \right|}$ with dimension |ε|, where c represents the total number of conductor segments (edges) in the grounding grid. The probability vector is represented as follows:


$$\begin{array}{c} P_{segment}=\text{Softmax}\left(GNN_{segment}\left(H,A\right)\right) \end{array}$$ (24)

Where $H\in R^{\left(\left|V\right|\times d\right)}$ is the node feature matrix, d denotes the feature dimension; $A\in R^{\left(\left|V\right|\times \left|V\right|\right)}$ represents the adjacency matrix; $GNN_{segment}$ is the graph neural network module dedicated to conductor segment classification, taking H and A as inputs and outputting fault scores for each conductor segment; $P_{segment}\in R^{\left|\nu \right|}$ is the conductor segment fault probability vector normalized via Softmax.

The second stage is precise location regression. Position regression uses a relative coordinate system, with the two endpoints of the conductor segment as references, to output the relative position parameters of the fault location:


$$\begin{array}{c} r_{fault}=GNN_{position}\left(H_{local},A_{local}\right) \end{array}$$ (25)

Where *r*_*fault*_∈[0,1] represents the relative position of the fault location on the conductor segment, and *H*_*local*_ and *A*_*local*_ represent the local node features and adjacency relationships centered on the candidate conductor segment, respectively. The final formula for calculating the location of the fault is follow:


$$\begin{array}{c} P_{fault}=P_{start}+r_{fault}\cdot (P_{end}-P_{start}) \end{array}$$ (26)

Where *P*_*start*_ and *P*_*end*_ represent the coordinate positions of the starting and ending points of the candidate conductor segment, respectively, while *P*_*fault*_ is the final predicted absolute fault position coordinate.

**Fig 4:**
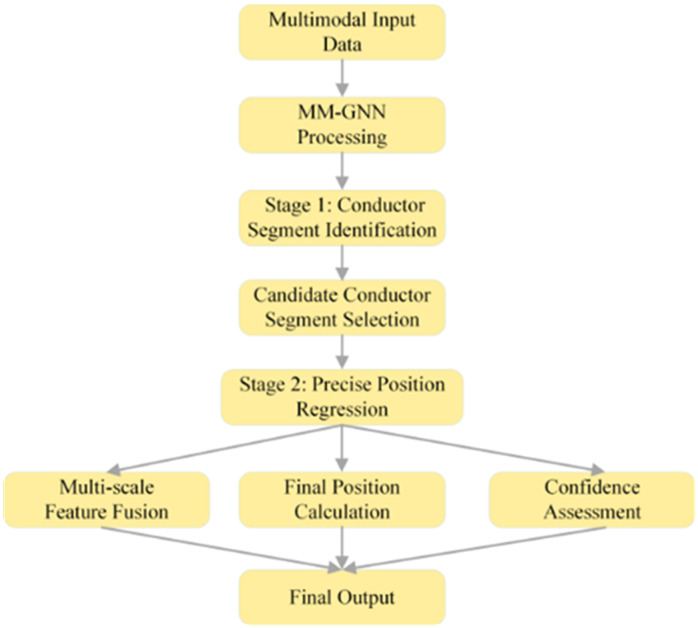
Conductor-level localization output mechanism flowchart.

Fig 4 depicts the overall process of conductor level positioning output. The model is based on multimodal inputs, and first outputs the fault probability vector *P*_*sement*_∈$R^{\left|\varepsilon \right|}$ (|ε| is the total number of conductor segments) for each conductor segment through *GNN*_*segment*_. Subsequently, *GNN*_*position*_ regresses the precise position of the candidate conductor segments. Finally, combining confidence evaluation to output fault coordinates, achieving end-to-end high-precision positioning.

## 4. Case verification analysis

### 4.1. Background of experimental setup

A simulation experiment is conducted based on the grounding grid of a 220kV substation in East China. The experiment used ANSYS Maxwell electromagnetic simulation software to establish a three-dimensional electromagnetic field model of the grounding grid, combined with MATLAB for data processing and algorithm implementation.

The main grid of the grounding grid simulation model is made of 50 mm × 5 mm galvanized flat steel, with a grid spacing of 15m × 20m and a coverage area of 180m × 120m. The vertical grounding electrode is made of 16 mm galvanized round steel, with a length of 2.5m, evenly arranged along the perimeter of the grid. The soil model is based on actual geological exploration data to establish a double-layer structure, with a surface soil resistivity of 120 Ω· m, a deep soil resistivity of 80 Ω· m, and a boundary depth of 3m.

The construction of fault samples follows the fault rules in the actual operation of the grounding grid. Based on historical fault statistical data analysis, four typical fault modes are experimentally designed. Conductor corrosion fault is simulated by increasing the conductor resistivity by 2–10 times. Loose connection fault introduces 0.1–2.0 Ω contact resistance at critical nodes. The conductor fracture fault is achieved by completely cutting off the conductor connection. The degradation fault of the grounding electrode will increase the contact resistance between the grounding electrode and the soil by 3–15 times. The Monte Carlo method is used to generate fault samples, with 8000 single fault samples, 2000 multiple fault concurrent samples, and 1000 normal state samples, ensuring sample diversity and representativeness.

Multimodal data acquisition is achieved by deploying virtual sensor networks at key nodes of the grounding grid. Electrical parameter measurement covers the potential, current density, and impedance characteristics of each testing point; Infrared thermal imaging data with a resolution of 640 × 480 pixels and a temperature measurement accuracy of ±0.1℃; The electromagnetic field distribution data is sampled in a 5m × 5m grid. Gaussian white noise is added to the data with a signal-to-noise ratio of 40-60dB. The experimental dataset is divided into training set, validation set, and testing set in a ratio of 7:2:1.

Model hyperparameters were optimized via grid search on the validation set. The graph convolutional network comprised three layers with feature dimensions of 128, 64, and 32 respectively; the multimodal fusion layer had an output dimension of 64. The Adam optimizer is employed with an initial learning rate of 0.001, adjusted using a cosine annealing strategy. Batch size is set to 32, and training is conducted for 200 epochs. Specifically, for the topological connectivity constraint loss weight *λ*_1_ and the spatial distance constraint loss weight *λ*_2_, separate searches are conducted on the validation set: *λ*_1_ is searched within the range of [0.5, 1.5] with a step size of 0.1, and *λ*_2_ is searched within the range of [0, 1.0] with a step size of 0.1. The search process comprehensively considers positioning errors and topological constraint violation rates. Finally, *λ*_1_ = 1.0 and *λ*_2_ = 0.5 are selected as the default parameter configurations for the model. α is searched within the range [0.5, 1.5] with a step size of 0.1, while β is searched within the range [0.1, 1.0] with a step size of 0.1. The final selection is α=1 and β=0.5, which achieves the best diagnostic accuracy (97.2%) and localization accuracy (1.28 m) on the validation set. The temporal learning module parameters are set as follows: time window length T = 48 hours (1-hour sampling interval), hidden layer dimensions of the two-layer LSTM structure set to 128 and 64 respectively, and window lengths for the multi-scale branches set to 12 hours, 48 hours, and 168 hours.

### 4.2. Comparison methods and evaluation indicators

#### 4.2.1. Method selection.

This section compares the Convolutional Neural Network based Multi source Fusion (CNN-MSF) [24], Long Short Term Memory based Multi source Fusion (LSTM-MSF) [25], Transformer based Multi source Fusion (Trans-MSF) [26] and standard Graph Neural Network (std-GNN) [27] methods with the methods proposed in the article. These baseline methods also adopt multimodal data fusion strategies and neural network architectures.

To ensure the fairness and reliability of the comparative experiments, all baseline methods are strictly consistent with the method proposed in this paper in terms of data processing, hyperparameter tuning, and evaluation criteria.

In terms of data consistency, all methods use exactly the same data preprocessing process (time alignment, spatial alignment, numerical standardization), the same data augmentation strategy (Gaussian noise injection, SNR = 40-60dB), and the same dataset partitioning (7:2:1). Except for Std-GNN, which only uses electrical parameter single modal data, all other methods use electrical, infrared, and electromagnetic modal data.

In terms of hyperparameter optimization, all baseline models are systematically optimized on the validation set through grid search, with the search range referring to the recommended settings and reasonable experience intervals in the original literature, and the optimal model configuration for validation set performance is reported. CNN-MSF adopts a 5-layer convolutional structure, with channel configurations of [64, 128, 128, 64, 32]. LSTM-MSF adopts a double-layer LSTM with a hidden layer dimension of 128. Trans MSF uses a 4-layer encoder and 8 attention heads; Std-GNN adopts 3-layer GCN with dimension configuration [128, 64, 32], which is consistent with the method proposed in this paper. All methods use the Adam optimizer (initial learning rate of 0.001), batch size of 32, training epochs of 200, and adopt the cosine annealing learning rate strategy.

In terms of training and evaluation, all methods use the same loss function configuration (classification loss is cross entropy, localization loss is mean square error, multitask weight 1:1), evaluate on the same test set, and use consistent evaluation metrics. Maintain consistent testing environment (servers equipped with high-performance GPUs). The performance differences between different methods only come from differences in model structure and fusion mechanism, rather than differences in implementation or training settings. The above consistency control ensures the fairness of comparative experiments, allowing performance differences to truly reflect the technical advantages of different methods.

#### 4.2.2. Construction of evaluation index system.

The fault recognition performance is evaluated using accuracy, recall, and *F*1 score [28,29]. The precision is defined as the ratio of the number of correctly identified fault samples to the total number of samples identified as faults, and its calculation formula is as follows:


$$\begin{array}{c} P=\frac{TP}{TP+FP} \end{array}$$ (27)

Where *TP* represents the true number of cases (correctly identified fault samples), and *FP* represents the false positive number of cases (incorrectly identified normal samples). The recall rate is defined as the ratio of the number of correctly identified fault samples to the total number of actual fault samples, and its calculation formula is expressed as follow:


$$\begin{array}{c} R=\frac{TP}{TP+FN} \end{array}$$ (28)

Where *FN* represents the number of false negative cases (unrecognized fault samples). The recall rate measures the algorithm’s ability to detect real faults, and a high recall rate indicates that the algorithm can effectively identify the vast majority of faults. The *F*1 score, as the harmonic mean of precision and recall, provides a comprehensive performance evaluation, and its calculation formula is:


$$\begin{array}{c} F1=\frac{2\times P\times R}{P+R} \end{array}$$ (29)

The *F*1 score takes into account both accuracy and recall, making it particularly suitable for evaluating the overall performance of fault detection tasks.

The accuracy of fault localization is evaluated based on the average localization error and localization success rate. Mean Absolute Error (MAE) is defined as the average Euclidean distance between the predicted fault location and the actual fault location, calculated using the following formula:


$$\begin{array}{c} MAE=\frac{\text{1}}{N}\sum_{i=1}^{N}\sqrt{(\overset{pred}{\underset{i}{x}}-\overset{true}{\underset{i}{x}}{)}^{\text{2}}+(\overset{pred}{\underset{i}{y}}-\overset{true}{\underset{i}{y}}{)}^{\text{2}}} \end{array}$$ (30)

Where *N* is the number of test samples, $\left(\overset{pred}{\underset{i}{x}},\overset{pred}{\underset{i}{y}}\right)$ is the predicted fault location of the *i*-th sample, and $\left(\overset{true}{\underset{i}{x}},\overset{true}{\underset{i}{y}}\right)$ is the actual fault location. The average positioning error directly reflects the positioning accuracy level of the algorithm, and the smaller the value, the more accurate the positioning. Accurate Success Rate (ASR) is defined as the proportion of samples with positioning errors less than a preset threshold, calculated using the following formula:


$$\begin{array}{c} LSR=\frac{\text{1}}{N}\sum_{i=1}^{N}𝕀(d_{i}\leq \tau ) \end{array}$$ (31)

Where *d*_*i*_ is the positioning error of the *i*-th sample, *τ* is the preset positioning accuracy threshold, and Ⅱ(•) is the indicator function.

### 4.3. Experimental results and analysis

#### 4.3.1. Analysis of different types of faults.

**Fig 5:**
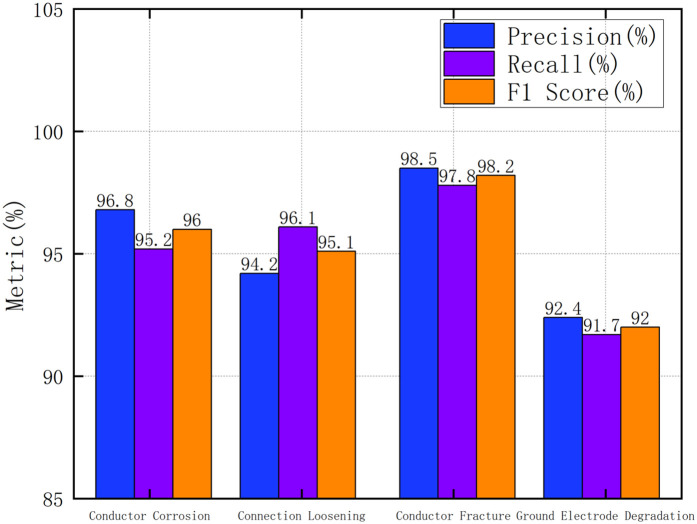
Comparison of the identification performance of different fault types.

Through the established evaluation index system, this section establishes detailed experimental methods to verify the effectiveness and applicability of four types of fault diagnosis and localization tasks. The faults exhibit different characteristic performances in terms of recognition performance, as shown in Fig 5.

The identification performance of conductor fracture faults has achieved the best results, with an accuracy rate of 98.5%, a recall rate of 97.8%, and an *F*1 score as high as 98.2%. This outstanding performance is due to the suddenness and severity of the fracture fault: a completely cut off current path produces drastic changes in electrical parameters, a significant temperature distribution anomaly is formed near the fracture point, and a large-scale electromagnetic field reconstruction is triggered.

As the most common progressive fault, conductor corrosion fault exhibits relatively balanced recognition performance, with an accuracy rate of 96.8%, a recall rate of 95.2%, and an *F*1 score of 96.0%. The gradual change in resistivity during the corrosion process causes abnormal electrical parameters, an increase in local current density leads to infrared temperature rise, and subtle but recognizable changes in electromagnetic field distribution.

The loose connection fault exhibits unique advantages in recognition performance, with an accuracy rate of 94.2%, a recall rate of up to 96.1%, and an F1 score of 95.1%. It is worth noting that although the accuracy is slightly lower than the first two types of faults, the recall rate is outstanding, indicating that this method has strong detection ability for loose faults and can effectively avoid the risk of missed detection.

Due to its unique physical mechanism and distributed impact characteristics, grounding electrode degradation faults face relatively significant challenges in identification. The accuracy rate is 92.4%, the recall rate is 91.7%, and the *F*1 score is 92.0%. Although it is relatively low among the four types of faults, it still maintains an acceptable level of recognition.

Table 2 provides a complete confusion matrix including normal states.

**Table 2:** Confusion matrix for fault diagnosis.

True categories\Predicted categories	Corrosion	Loose connection	Fracture	Deterioration of grounding electrode	Normal	Recall (%)
Corrosion	209	5	3	2	1	95.0
Loose connection	4	211	2	2	1	95.9
Fracture	2	2	215	0	1	97.7
Deterioration of grounding electrode	6	5	1	202	6	91.8
Normal	0	0	0	1	99	99.0
Column total	221	223	221	206	109	980
Accuracy(%)	94.6	94.6	97.3	98.1	91.7	

The test set consists of 980 samples, of which the four types of faults each contain 220 samples in the true category dimension (totaling 89.8%), and the normal state contains 100 samples (accounting for 10.2%). In Table 2, rows represent real categories and lists represent predicted categories. Diagonal elements represent the number of correctly classified samples, while non diagonal elements represent the number of misclassified samples. At the end of the line, recall rates for each category were provided, with an overall accuracy of 95.6%.

The confusion matrix reveals the detailed classification performance and main misclassification patterns of the model. Diagonal elements dominate, with a recall rate of over 91.8% for all categories and an overall accuracy rate of 95.6%, consistent with the performance shown in Fig 5. The main misclassification patterns include: there is a small amount of bidirectional confusion between corrosion and loosening (5 cases and 4 cases, each about 2%), which is due to similar early manifestations of both – both leading to increased local resistance and temperature rise, which need to be distinguished by temporal features; The degradation of grounding electrodes is most easily misjudged as corrosion (6 cases, 2.7%) or normal state (6 cases, 2.7%), reflecting the distributed characteristics and concealment of degraded faults; The least misclassification of conductor fractures (5 cases, 2.3%) confirms that their significant features are easy to identify. Analyze the mixing phenomenon of corrosion and loosening from two dimensions: model feature extraction ability and misclassified sample features. Model feature extraction: MM-GNN mainly relies on three types of discriminative information - ① Temporal evolution: corrosion resistance increases monotonically and gradually (within 48 hours), while loosening resistance fluctuates or changes intermittently. ② Spatial diffusion: Corrosion temperature anomalies are continuously distributed along the conductor segment, and loose overheating is concentrated at a single node. ③ Electromagnetic field: loosening causes sudden changes in local current density, sharp peaks in magnetic field gradient at fault nodes, and smooth changes in corrosion. Characteristics of misclassified samples: 9 cases of misclassification (5 cases of corrosion→loosening, 4 cases of loosening→corrosion) all showed a mild degree of fault (corrosion resistivity increased by 2−3 times, loose contact resistance was 0.1–0.3 Ω, all close to the simulation lower limit). In the early stages of the fault, the temporal characteristics are not significant, making it difficult for LSTM to make reliable judgments. The infrared temperature rise is below 1 ℃, approaching the accuracy boundary of the thermal imager (±2℃), and the advantage of multimodal fusion is weakened. The above analysis indicates that the confusion between corrosion and loosening is mainly due to the high overlap of mild faults in the feature space, rather than model structural defects.

The category imbalance analysis shows that although there is a category imbalance ratio of about 8.8:1 (fault 880 vs normal 100), the model does not exhibit excessive skewness towards the majority of categories. The performance of all four types of faults remains at a high level (accuracy of 94.6% −98.1%, recall of 91.8% −97.7%), verifying the effectiveness of the weighted cross entropy loss function – normal state samples are given higher weights to ensure that the model fully focuses on minority classes. The overall accuracy of 95.6% indicates that the model has excellent performance in fault diagnosis tasks.

**Fig 6:**
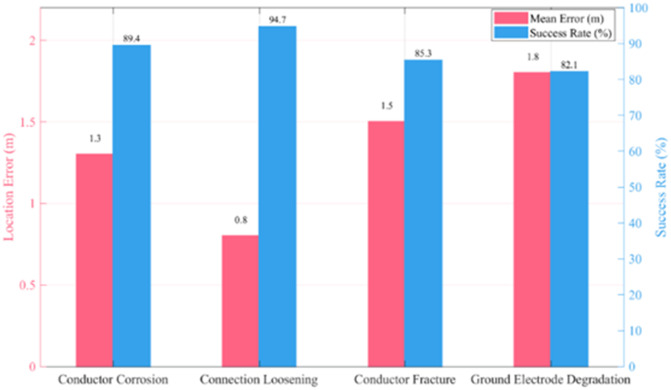
Comparison of location performance of different fault types.

Fig 6 shows the differences in localization accuracy for different types of faults, and the localization characteristics of each type of fault are closely related to their physical mechanisms.

The loose connection fault exhibits the best positioning performance, with an average positioning error of only 0.8 meters and a positioning success rate of up to 94.7%. This excellent positioning accuracy is due to the node concentration characteristic of loose faults: the impact of faults is mainly limited to specific connection points and their direct neighborhoods, providing favorable conditions for accurate positioning.

The localization performance of conductor corrosion faults is moderate, with an average localization error of 1.3 meters and a localization success rate of 89.4%. The main challenge in locating corrosion faults lies in their asymptotic and spatial continuity characteristics, where the corrosion area often has a certain diffusion range rather than a single point of failure.

The average positioning error of conductor fracture faults is 1.5 meters, and the success rate of positioning is 85.3%. The success rate of locating degraded grounding faults is 82.1%, reflecting the distributed characteristics of degraded faults and the ambiguity of localization targets. Multimodal information fusion can identify the main areas affected by degradation by comprehensively analyzing changes in overall grounding impedance, abnormal heat dissipation patterns, and changes in current leakage paths.

**Fig 7:**
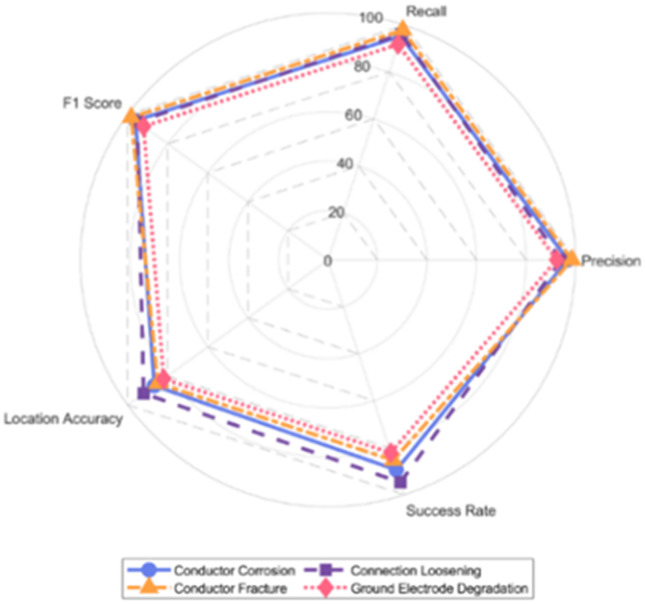
Radar chart of the comprehensive performance of fault types.

The comprehensive performance radar chart in Fig 7 visually displays the balanced performance of four types of faults on various performance indicators. From the radar chart, it can be observed that the loose connection fault performs outstandingly in positioning related indicators, forming a relatively balanced performance profile. Conductor fracture faults have a significant advantage in identifying relevant indicators, but the positioning indicators are relatively weak. The conductor corrosion fault exhibits a relatively balanced comprehensive performance, and the development of various indicators is relatively coordinated. Although the performance indicators of the grounding electrode degradation fault are relatively low, it still maintains an acceptable level of performance.

**Fig 8:**
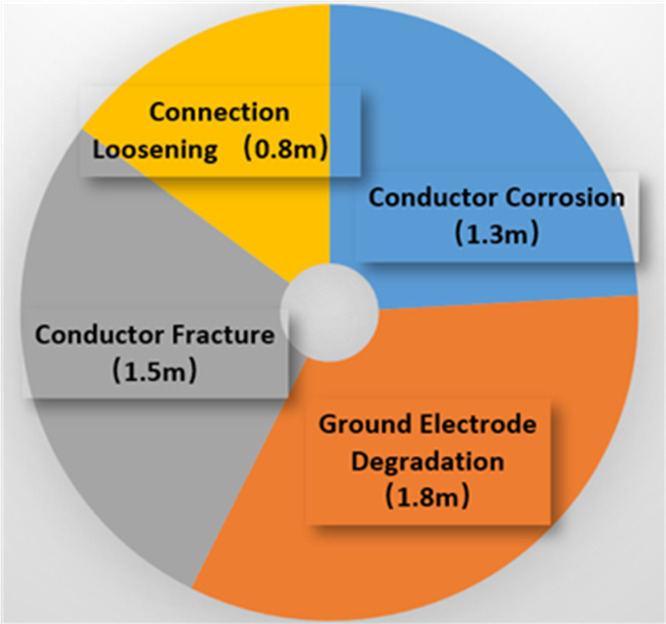
Average positioning error distribution.

**Fig 9:**
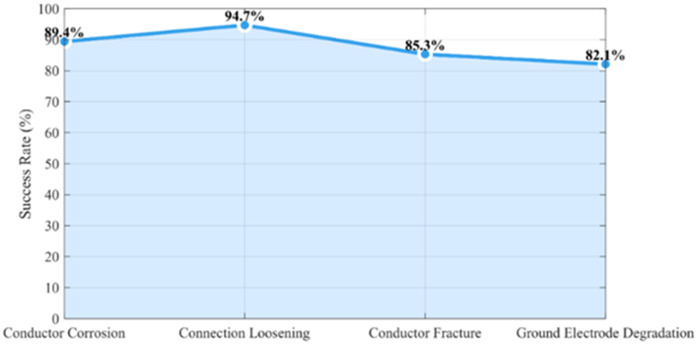
Comparison of targeting success rates.

The distribution of positioning errors in Fig 8 further reveals the differences in positioning characteristics of various types of faults. The minimum error of loose connection faults (0.8 meters) reflects its advantage in locating concentrated faults; The maximum error of grounding electrode degradation fault (1.8 meters) reflects the inherent challenge of distributed fault location. The comparison of localization success rates in Fig 9 shows that the success rate for loose connection faults is the highest (94.7%), while the success rate for ground electrode degradation faults is the lowest (82.1%), which is closely related to the physical characteristics and impact range of various types of faults.

Based on the physical characteristics and impact modes of different types of faults, the experimental results show that the proposed method can adaptively adjust the fusion strategy of multi-source information. For sudden faults with obvious characteristics (such as conductor breakage), algorithms rely more on drastic changes in electrical parameters for identification. Gradual faults (such as conductor corrosion) require algorithms to focus more on the temporal variation patterns of multimodal information. For local faults (such as loose connections), the algorithm fully utilizes topological neighborhood information to achieve precise localization. Distributed faults (such as ground electrode degradation), the algorithm identifies the affected area through global feature analysis.

Based on the above analysis, the average *F*1 score for identifying the four types of faults reached 95.3%, the average positioning error was controlled within 1.4 meters, and the positioning success rate exceeded 87.9%.

#### 4.3.2. Analysis of ablation experiment.

This section presents a detailed ablation experiment to analyze the contribution of each module to the overall fault diagnosis and localization performance.

(1) Network variants with different configurations

**Fig 10:**
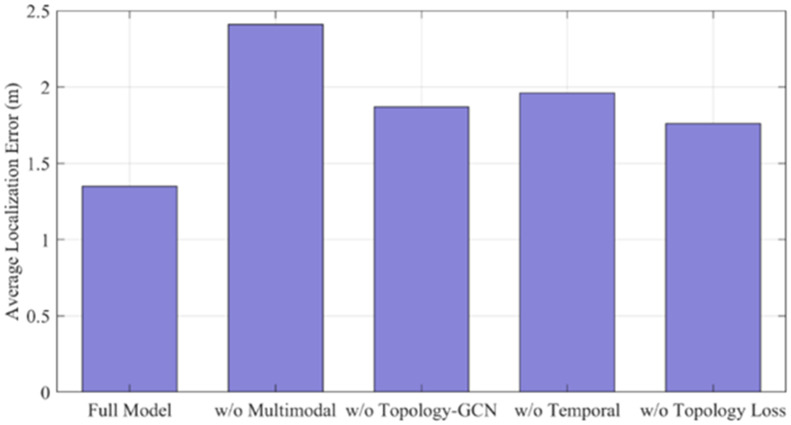
Comparison of fault location accuracy for different network configurations.

**Fig 11:**
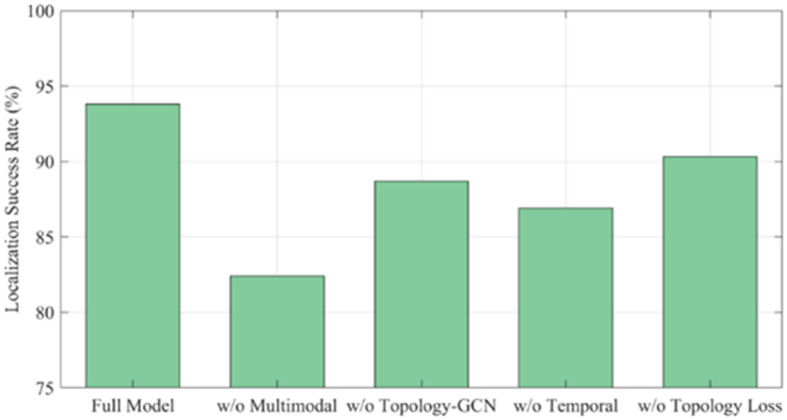
Comparison of fault location success rates for different network configurations.

This section constructs five different configurations of network variants for ablation analysis: a complete MM-GNN model (Full Model), a single modal graph neural network with removed multimodal fusion module (w/o Multimodal), a network with removed topological graph convolution design using standard graph convolution (w/o Topology GCN), a static network with removed temporal dynamic learning mechanism (w/o Temporal), and a network with removed topological constraint loss function (w/o Topology Loss). Each variant is trained and tested under the same experimental conditions to ensure the objectivity and comparability of ablation experimental results. Figs 10 and 11 show the performance comparison of different network configurations in terms of fault location accuracy.

From the average positioning error results in Fig 10, it can be clearly observed that the complete MM-GNN model performs the best in all indicators, with an average positioning error of only 1.35 meters, significantly better than other variants. After removing the multimodal fusion module, the positioning accuracy of the network significantly decreased, and the average error increased to 2.41 meters. Single mode information often has limitations, while multimodal fusion can fully exploit the complementarity between different data sources, so as to obtain more comprehensive and accurate fault feature representation. The success rate comparison in Fig 11 further confirms this conclusion, with the complete model achieving a localization success rate of 93.8%, while the success rate drops to 82.4% after removing multimodal fusion.

In Fig 10, when using standard graph convolution instead of specially designed topology graph convolution, the positioning accuracy of the model showed significant degradation, with an average error increase to 1.87 meters. The standard graph convolution operation cannot fully capture the physical connection characteristics and electrical propagation laws of conductor segments in the grounding grid. Correspondingly, Fig 11 shows that its localization success rate has decreased from 93.8% of the complete model to 88.7%.

**Fig 12:**
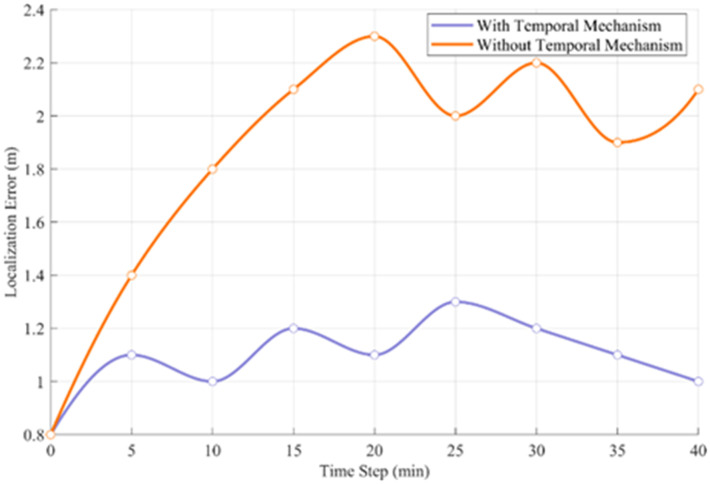
Comparison of the effects of the time-series dynamic learning mechanism.

The ablation experiment results of the temporal dynamic fault feature learning mechanism are shown in Fig 12. After removing this module, the network can only handle static fault characteristics and cannot capture dynamic change patterns during the fault development process, resulting in an increase in positioning error to 1.96 meters.

When the topological constraint loss function is removed, although the network can still perform fault localization, the physical rationality of the localization results significantly decreases, and the average error increases to 1.76 meters. More importantly, unconstrained networks may produce positioning results that violate the topology of the grounding network in certain situations.

The ablation experiment also revealed the differences in contributions of different modules under different types of faults. The contribution of the multimodal fusion module is most significant for corrosion related faults, as the corrosion process involves the comprehensive manifestation of multiple physical phenomena. The role of topological graph convolution is more prominent in fault types, as faults directly affect the topological connectivity of the grounding network. The timing dynamic learning mechanism plays a key role in the occurrence of poor contact faults, as these faults are often accompanied by intermittent changes in electrical parameters.

(2) Analysis of module contribution differences under different fault types

This section analyzes the performance contributions of each module under four typical fault categories. Table 3 shows the incremental localization error for different fault types after removing specific modules.

**Table 3:** Incremental analysis of module positioning errors under different fault types.

Remove Module	Conductor corrosion	Loose connection	Conductor break	Grounding electrode deterioration
w/o Multimodal	+1.42	+0.89	+1.15	+1.68
w/o Topology-GCN	+0.63	+0.48	+0.95	+0.41
w/o Temporal	+0.85	+0.52	+0.48	+0.73
w/o Topology Loss	+0.38	+0.51	+0.56	+0.47

Grounding electrode degradation faults exhibit the strongest dependency on the multimodal fusion module (error increment +1.68 meters), significantly higher than other fault types. The physical reason for this phenomenon lies in the nature of grounding electrode degradation: it primarily manifests as increased contact resistance between the grounding electrode and soil. This impact is dispersed and subtle, often causing single-modal signals to be overwhelmed by environmental noise, making it difficult to form clear distinguishing features. In contrast, loose connection faults exhibit relatively lower dependency (+0.89 meters). This is because the sudden increase in contact resistance caused by loosening (typically reaching 0.5–2.0 Ω) is highly evident in electrical parameters, resulting in significant gradient anomalies in node potential. A single electrical modality already possesses strong discrimination capability. Conductor corrosion (+1.42 m) and conductor breakage (+1.15 m) exhibit moderate contribution values, though their underlying physical mechanisms differ.

Differentiated Contributions of the Topological Graph Convolution Module. Conductor break faults exhibit the most pronounced dependence on topological graph convolution (+0.95 meters), significantly higher than other fault types. This arises because breaks directly alter the topological connectivity of the grounding network, triggering global current redistribution. The topology-aware graph convolutional approach introduced in this paper incorporates physical properties like conductor resistance to more precisely predict current redistribution paths. Conversely, grounding electrode degradation faults exhibit the lowest dependency (+0.41 meters). This is because degradation primarily affects local node-to-ground leakage characteristics rather than internal grid topological connections. Identifying such faults relies more on node-specific features than neighborhood aggregation information. Contributions from loose connections (+0.48 m) and conductor corrosion (+0.63 m) are moderate, indicating that while these faults also involve topological relationships, they depend more on the precise representation of local features.

Differentiated Contributions of Temporal Dynamic Learning Mechanisms. Conductor corrosion faults exhibit the highest demand for temporal learning mechanisms (+0.85 m), fully consistent with corrosion’s physical nature as a prototypical progressive failure. Typically spanning months or even years, corrosion-induced resistivity changes demonstrate a pronounced monotonically increasing trend within this study’s 48-hour monitoring window. The LSTM network effectively captures these long-term dependencies, predicting corrosion progression from historical data to identify emerging faults before they fully manifest. Grounding electrode degradation also exhibits strong temporal dependence (+0.73 meters). In contrast, conductor breaks (+0.48 m) and loose connections (+0.52 m) exhibit weaker temporal dependencies as sudden or intermittent failures. Breaks typically occur rapidly, limiting the value of temporal information; while loose connections may show intermittent characteristics, their primary identification relies on instantaneous electrical parameter anomalies rather than long-term evolution trends.

Differentiated Contributions to Topological Constraint Loss. The dependency of various faults on topological constraint loss is relatively balanced (0.38–0.56 m), indicating this constraint holds universal value in ensuring the physical plausibility of localization results. However, subtle differences remain observable: conductor breakage faults exhibit slightly higher dependency (+0.56 m), as current redistribution post-breakage readily causes localization results to deviate from the actual conductor path. By restricting solutions to lie on conductor segments, the topology constraint effectively corrects this deviation. Loose connections (+0.51 m) also contribute significantly for similar reasons: complex potential gradient distributions near loose points may yield multiple local extrema, and the topology constraint guides the model toward reasonable positions at conductor junctions. The dependencies for corrosion (+0.38 m) and ground electrode degradation (+0.47 m) are relatively low. This is because the affected areas for these two fault types typically align closely with the physical locations of the conductor or ground electrode. Consequently, even without explicit topology constraints, the model rarely locates faults in non-conductor regions.

Analysis of Module Synergy Effects. Further experiments reveal nonlinear synergistic enhancement effects among modules. When both multimodal fusion and topological graph convolution are removed simultaneously, the localization error for conductor break faults increases by 2.38 meters—exceeding the sum of their individual contributions (1.15 + 0.95 = 2.10 meters). This indicates that topological graph convolution more effectively propagates and integrates features from multimodal fusion, yielding a synergistic effect where the whole is greater than the sum of its parts. For grounding electrode degradation faults, the synergistic effect between multimodal fusion and temporal learning was most pronounced; simultaneous removal resulted in an error increase of 2.67 meters (>1.68 + 0.73 = 2.41 meters).

**Fig 13:**
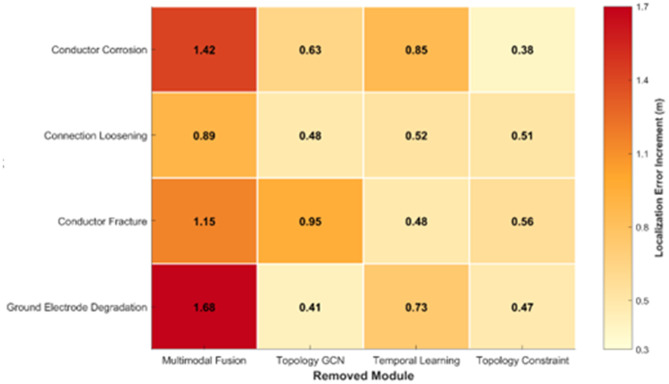
Module contribution differences under different fault types (incremental positioning error).

Targeted Summary of Module Design. The aforementioned differential analysis fully demonstrates the targeted nature of each innovative module design in this paper: (1) The multimodal fusion module is particularly effective for concealed and dispersed faults (grounding electrode degradation +1.68 meters), with its adaptive weighting mechanism dynamically adjusting the contribution of each modality based on signal quality. (2) The Topology Map Convolution Module targets faults affecting global topological connections (conductor breakage + 0.95m). Its physically-aware neighborhood aggregation accurately models current redistribution. (3) The Temporal Learning Mechanism specifically captures the evolution patterns of progressive faults (corrosion + 0.85m, degradation + 0.73m). Its multi-scale design accommodates varying fault progression rates. (4) The topology-constrained loss serves as a universal constraint, ensuring the physical feasibility of locating all fault types. Its corrective effect is particularly pronounced in complex current distribution scenarios (breakage +0.56 m, loosening +0.51 m). These findings provide theoretical guidance for customized diagnostic strategies tailored to different fault types and further validate the rationality and effectiveness of the MM-GNN architecture design. Fig 13 visually illustrates this differentiated contribution pattern through a heatmap.

Fig 13 illustrates the incremental localization error for various fault types after removing different modules. Darker colors indicate greater importance of the module for that fault type. Key findings: (a) Ground electrode degradation exhibits the strongest dependence on multimodal fusion (1.68 m), as its weak characteristics require multi-source collaborative confirmation; (b) Conductor breakage exhibits significant dependence on topology graph convolution (0.95 m), necessitating modeling of global current redistribution; (c) Conductor corrosion demands the highest requirement for temporal learning (0.85 m), requiring capture of gradual evolutionary patterns; (d) Topology constraint loss remains relatively balanced across fault types (0.38–0.56 m), ensuring physically plausible localization.

#### 4.3.3. Robustness analysis of multimodal fusion.

This section designs a systematic experimental analysis of the sensitivity of model performance to different modal combinations and data quality.

Table 4 shows the performance comparison under different modal combinations. Complete three modes achieve 96.8% accuracy and 1.23m positioning error. When a single modality is missing, the electrical and infrared combination has the best performance (94.5%, 1.58 meters), while the infrared and electromagnetic combination has the worst performance (88.7%, 2.35 meters), highlighting the core position of electrical parameters. Single modal experiments have shown that using only electrical parameters can achieve an accuracy of 91.3%, while infrared (82.4%) or electromagnetic (79.6%) alone are difficult to meet engineering requirements, verifying the necessity of multimodal fusion.

**Table 4:** Model Performance under Different Modal Combinations.

Modal combination	Diagnostic accuracy	Positioning error (m)
Electrical+Infrared+Electromagnetic	96.8%	1.23
Electrical+Infrared	94.5%	1.58
Electrical+Electromagnetic	93.2%	1.72
Electrical	91.3%	1.89
Infrared+Electromagnetic	88.7%	2.35
Infrared	82.4%	3.12
Electromagnetic	79.6%	3.45

Table 5 shows that when the noise level increases to SNR = 20dB, the adaptive fusion mechanism automatically reduces the weight of the contaminated mode (electromagnetic field) to 0.13, and increases the weight of the stable mode (electrical, infrared) to 0.52 and 0.35. This dynamic adjustment reduces performance degradation by about 50% (accuracy 88.4% vs fixed weight 82.1%), verifying the effectiveness of the adaptive mechanism.

**Table 5:** Model robustness under different noise levels.

SNR (dB)	Accuracy	Error (m)	Electrical weight	Infrared weight	Electromagnetic weight
Noiseless ∞	96.8%	1.23	0.42	0.26	0.32
Low noise 40	95.2%	1.35	0.43	0.28	0.29
Medium noise 30	92.7%	1.62	0.48	0.31	0.21
High noise 20	88.4%	2.18	0.52	0.35	0.13

The infrared weight is the highest (0.38) in conductor corrosion faults due to significant temperature rise characteristics. The electrical weight is the highest in loose connection faults (0.52), due to a significant increase in contact resistance. The weight distribution of conductor fracture and ground electrode degradation is relatively balanced, reflecting the need for multimodal collaborative confirmation. There are significant differences in the degree of influence of mode loss on different fault types: the connection looseness can reach 92.1% accuracy only by using electrical mode, while the performance of any mode of grounding electrode degradation loss is significantly reduced, which is highly consistent with the fault physical characteristics.

#### 4.3.4. Sensitivity analysis of topological constraint loss weights.

This paper conducted a systematic sensitivity analysis experiment on *λ*_1_ and *λ*_2_. In the experiment, fix one parameter as the optimal value, change the value of the other parameter, and evaluate the physical rationality (topological constraint violation rate) of the model’s conductor level positioning error and prediction results on the validation set. Table 6 presents detailed sensitivity analysis results.

**Table 6:** Sensitivity analysis of topological constraint loss weight parameters.

*λ*_1_	*λ*_2_	Average positioning error	Topology violation rate	Localization success rate	Explanation
0.5	0.5	1.58 meters	4.2%	90.2%	*λ*_1_ is too small, with insufficient constraints
0.7	0.5	1.45 meters	1.8%	91.8%	Decreased violation rate
0.9	0.5	1.38 meters	0.3%	93.2%	Approaching optimal
1.0	0.5	1.35 meters	0	93.8%	Optimal combination
1.2	0.5	1.42 meters	0	93.0%	Constraints are starting to become too strong
1.5	0.5	1.53 meters	0	92.1%	Excessive constraints result in decreased accuracy
1.0	0.2	1.48 meters	0	92.3%	*λ*_2_ slightly small
1.0	0.3	1.41 meters	0	92.9%	*λ*_2_ smaller
1.0	0.7	1.39 meters	0	93.3%	*λ*_2_ slightly large
1.0	0.9	1.46 meters	0	92.5%	*λ*_2_ is too large and excessively smooth

The experimental results indicate that the weight parameters in the topological constraint loss have a significant impact on the conductor level localization performance of the model. As the topological connectivity constraint weight *λ*_1_ increases, the model’s constraint on the physical topology structure of the grounding network gradually strengthens. When the value of *λ*_1_ is small (such as *λ*_1_ = 0.5). The topological constraint violation rate reaches 4.2%, and the average positioning error is 1.58 meters. As *λ*_1_ increases, the topological consistency significantly improves, the violation rate rapidly decreases, and zero topological violation rate is achieved at *λ*_1_ = 1.0, while achieving a minimum average positioning error of 1.35 meters, indicating that this value achieves the best balance between positioning accuracy and physical constraints. However, as *λ*_1_ further increases, overly strong topological constraints begin to limit the model’s ability to express complex fault modes, resulting in a decrease in localization accuracy.

The spatial distance constraint weight *λ*_2_ mainly affects the spatial consistency of the predicted positions of adjacent conductor segments. When the value of *λ*_2_ is small (such as *λ*_2_ = 0.2), the spatial constraint effect is weak, resulting in an increase in positioning error to 1.48 meters; As *λ*_2_ increases, spatial consistency gradually strengthens and optimal performance is achieved at *λ*_2_ = 0.5. As *λ*_2_ continues to increase, overly strong smoothing constraints will weaken the model’s ability to accurately characterize local fault locations.

Further sensitivity analysis shows that within the reasonable range of *λ*_1_∈[0.9, 1.1] and *λ*_2_∈[0.4, 0.6], the model performance remains relatively stable, with an average positioning error variation of less than 0.1 meters, verifying the robustness of the selected parameter configuration. Taking into account factors such as positioning accuracy, physical rationality, and spatial consistency, we ultimately selected *λ*_1_ = 1.0 and *λ*_2_ = 0.5 as the default parameter settings for the model.

#### 4.3.5. Overall performance analysis.

The calculations in Table 7 indicate that the MM-GNN method proposed in this paper demonstrates significant technical advantages in all key performance indicators. From the perspective of fault diagnosis accuracy, the MM-GNN method achieved 96.8%, which is 3.6 percentage points higher than the best performing baseline method Trans MSF (93.2%), and the improvement is more significant compared to other baseline methods. The diagnostic accuracy of the CNN-MSF method is 89.4%, mainly due to the inherent limitations of convolutional neural networks in handling non Euclidean topological structures of grounding networks; Although the LSTM-MSF method has advantages in temporal feature processing, its 91.7% accuracy indicates a lack of effective modeling of spatial topological relationships; Although the Std GNN method considers the structural characteristics of the diagram, its accuracy rate is only 87.3%, which is the worst among all methods, because only single modal information is used.

**Table 7:** Overall performance comparison of different methods.

M	DA (%)	PE(m)	CT(s)	CE
CNN-MSF	89.4	4.67	3.1	Average
LSTM-MSF	91.7	3.82	3.7	Average
Trans-MSF	93.2	2.95	1.8	Good
Std-GNN	87.3	2.34	4.2	Average
MM-GNN	96.8	1.23	2.3	Optimal

Notes: Methods(M), Diagnostic accuracy(DA), Position error(PE), Calculate time(CT), Comprehensive evaluation(CE).

In terms of fault location accuracy, the MM-GNN method proposed in this paper achieved an average positioning error of 1.23 meters, successfully meeting the precise positioning requirements at the conductor level. The positioning error of CNN-MSF method is as high as 4.67 meters, which is mainly due to the difficulty of convolution operation in effectively handling the complex topology structure of the grounding network; The positioning error of the LSTM-MSF method is 3.82 meters. Although it has improved compared to CNN-MSF, it still cannot meet the engineering requirements for precise positioning; The Trans MSF method reduces the positioning error to 2.95 meters through attention mechanism, but due to the lack of consideration for the inherent structural characteristics of the grounding network, there is still significant room for improvement in its positioning performance; The positioning error of Std GNN method is 2.34 meters, which verifies the important role of graphic structure modeling in improving positioning accuracy, but the limitation of single mode information makes it impossible to further optimize.

From the perspective of adaptive fusion mechanism, this method is compared and analyzed with existing baselines. The optimal baseline Trans MSF (Transformer Self Attention) has a simulation accuracy of 93.2% and a localization accuracy of 2.95m. However, the disadvantage is that the attention weights are uniformly calculated at the batch level and cannot distinguish modal quality differences between samples. In on-site testing, the accuracy rate decreased by 4.7 percentage points (→ 88.5%). This article MM-GNN: simulation accuracy of 96.8% (higher starting point), on-site accuracy of 91.3% (absolute value still better than 88.5%), attenuation of 5.5 percentage points; The incremental positioning error is only + 0.64m, significantly better than Trans MSF (+0.73m) and other baselines (+0.87-1.15m), with a comprehensive robustness rating of Mild (Best). The advantage comes from sample adaptive fusion: under SNR = 20dB noise, the weight of contaminated electromagnetic fields is automatically reduced to 0.13, improving stable modes (electrical 0.52, infrared 0.35), and reducing performance degradation by about 50% compared to fixed weight strategies (88.4% vs 82.1%). Single mode baseline Std GNN: only electrical parameters, with an accuracy rate of 87.3% and a positioning error of 2.34m, there is a significant gap with the three mode synergy in this paper (the accuracy rate is 9.5% lower and the error is 1.11m higher), which verifies the necessity of multi-source fusion. Based on the above analysis, this method outperforms existing adaptive fusion baselines in three dimensions: fusion granularity (sample level vs batch level), physical constraint embedding (explicit Ohm’s law vs pure data-driven), and localization robustness under sensor degradation.

Based on the overall analysis of the three key performance indicators in Table 7, the proposed MM-GNN method demonstrates comprehensive technical advantages in the task of grounding grid fault diagnosis and localization. This method not only achieved the highest diagnostic accuracy of 96.8%, but also achieved the minimum positioning error of 1.23 meters. At the same time, it maintained a good response time of 2.3 seconds in terms of computational efficiency, achieving the optimal balance between accuracy and efficiency. The comprehensive performance fully validates the effectiveness of the three key technological innovations proposed in this paper: the topology graph convolution design guided by the grounding grid effectively captures the spatial structural characteristics of the grounding grid, the multimodal information adaptive fusion strategy fully explores the complementary value of multi-source data, and the topology constraint loss function ensures the engineering rationality of the positioning results.

#### 4.3.6. Field data validation and robustness analysis.

This study conducted field data acquisition and verification at two operational substations.

(1) On-site Testing Environment and Data Acquisition

This study selected two representative substations for field testing. Test Site A: A 220kV substation locates in a coastal area with 15 years of operation. The site exhibits distinct multi-layered soil structure: the surface layer has high salt content with resistivity ranging from 30 to 80 Ω·m, while the deeper layer consists of sandy soil with resistivity between 150 and 200 Ω·m, demonstrating pronounced soil stratification. The grounding grid, after prolonged operation, exhibits multiple corrosion points and aged connections. The grid covers an area of approximately 160m × 100m, employing 50 mm × 5 mm galvanized flat steel for the main grid with a spacing of 12m × 15m. Test Site B: An 110kV substation located within an industrial park, with 8 years of operational history. Large power electronic equipment and frequency conversion devices are distributed around this site, with external electromagnetic interference intensity reaching 15–25 dBμV/m—significantly exceeding the 5 dBμV/m baseline value set in the simulation environment. Soil resistivity is relatively uniform at 90–120 Ω·m. The grounding grid covers an area of approximately 120m × 80m, with the main grid constructed using 40 mm × 4 mm galvanized flat steel. On-site data collection employed portable testing equipment, specifically: Electrical parameter measurements were taken using a ground resistance tester (Model ZC-8, accuracy ±2%) at 36 test points per site to collect ground resistance and node potential data; Infrared thermal imaging was performed using a portable thermal imager (Model FLIR E60, resolution 320 × 240, temperature accuracy ±2°C) to scan exposed grounding down conductors and suspicious areas; Electromagnetic field distribution measurements employed a magnetic field detector (Model HI-3604) to measure magnetic flux density at a 2m × 2m grid spacing on the ground surface. The time synchronization of the three types of sensors is achieved through the following scheme: using the built-in clock of the electrical parameter tester as the main clock reference, before starting the collection at each testing point, the operator sends a unified start command through the intercom. The infrared thermal imager and magnetic field detector trigger the collection within 1 second after receiving the command, and each device records the local timestamp. In the data post-processing stage, the three types of data are aligned to a unified sampling time grid (based on the electrical parameter 1 Hz sampling interval) according to the timestamps of each device, with a time alignment error of no more than 1 second, meeting the engineering requirements for synchronization accuracy in power frequency fault diagnosis. 52 failure samples are confirmed, and it contains 18 corrosion failures, 12 loose connections, 9 degraded grounding electrodes, and 13 concurrent multiple failures. Site A accounted for 38 samples (primarily corrosion and degradation), while Site B had 14 samples (mainly loose connections).

(2) On-site Data Validation Results

On 52 field failure samples, the overall performance of the MM-GNN method is as follows.

As shown in Table 8, the proposed method achieves the highest diagnostic accuracy (91.3%) and the smallest positioning error (1.87 meters) on field data, significantly outperforming all baseline methods. Considering the relatively limited scale of on-site testing, the above results preliminarily indicate that the proposed method has good applicability in real engineering environments, and further verification is needed in larger scale on-site scenarios in the future. Regarding positioning success rate, it reaches 86.5% under a 2-meter accuracy requirement and 94.2% under a 3-meter accuracy requirement, meeting practical engineering needs. To more clearly evaluate the impact of field environments on performance, Table 9 compares the performance differences of each method on simulated data versus field data.

**Table 8:** Comparison of on-site data validation results.

Method	Diagnostic accuracy (%)	Positioning error (m)	Success Rate ≤2m (%)	Success Rate ≤ 3m (%)
CNN-MSF	82.7	2.65	71.2	82.7
LSTM-MSF	84.6	2.48	75.0	86.5
Trans-MSF	88.5	2.12	80.8	90.4
Std-GNN	80.8	2.89	67.3	80.8
MM-GNN	91.3	1.87	86.5	94.2

**Table 9:** Comparison of on-site data validation results.

Method	Diagnostic accuracy (%)		Positioning error (m)		Performance Degradation Assessment
	Simulation	On-site	Simulation	On-site	
CNN-MSF	89.4	82.7(−6.7)	4.67	2.65(+1.15)	Relatively Severe
LSTM-MSF	91.7	84.6(−7.1)	3.82	2.48(+1.13)	Relatively Severe
Trans-MSF	93.2	88.5(−4.7)	2.95	2.12(+0.73)	Moderate
Std-GNN	87.3	80.8(−6.5)	2.34	2.89(+0.87)	Severe
MM-GNN	96.8	91.3(−5.5)	1.23	1.87(+0.64)	Mild (Best)

Table 9 clearly demonstrates the robust advantages of the proposed method: compared to simulation conditions, performance degradation on real-world data is minimal (accuracy decreases by only 5.5%, while positioning error increases by just 0.64 meters), whereas the baseline methods exhibit accuracy drops of 6.5–7.1% and positioning errors increasing by 0.73–1.15 meters. This validates the effectiveness of multimodal adaptive fusion and topological physical constraints in addressing real-world environmental challenges.

(3) Performance Comparison Under Different Environmental Conditions

To conduct an in-depth analysis of the impact of different environmental factors, this study performed a detailed comparative performance analysis of the two test sites. Table 10 Shows the performance differences under conditions of soil non-uniformity.

**Table 10:** Performance comparison under different soil conditions.

Test Site	No. of Faults	Diagnostic accuracy (%)	Positioning error (m)	Major Environmental Characteristics
Site A (220 kV)	38	89.5	2.06	Multi-layer soil; resistivity 30–200 Ω·m;15 years in operation
Site B (110 kV)	14	92.9	1.52	Relatively uniform soil; resistivity 90–120 Ω·m; 8 years in operation
Site A (with soil stratification prior)	38	92.1	1.89	Introduce prior information on soil stratification

As shown in Table 10, Site A exhibits relatively lower performance (89.5% accuracy, 2.06-meter error) due to its complex multi-layered soil structure, while Site B demonstrates superior performance (92.9% accuracy, 1.52-meter error) under relatively homogeneous soil conditions. After incorporating prior knowledge of soil stratification, Site A’s performance significantly improved (accuracy increased to 92.1%, error reduced to 1.89 meters), demonstrating the model’s strong scalability and adaptability. Table 10 presents the robustness test results under electromagnetic interference conditions.

The results in Table 11 indicate that although strong electromagnetic interference affects performance, the multimodal fusion strategy demonstrates excellent interference resistance. During high-interference periods, the average weight of the electromagnetic field modality decreased from 0.32 during low-interference periods to 0.15, while the weight of the electrical parameter modality increased from 0.42 to 0.54, and that of the infrared thermal imaging modality rose from 0.26 to 0.31. This automatic weight adjustment mechanism enables the model to maintain performance by relying on other reliable modalities when certain modalities are severely disrupted.

**Table 11:** Performance comparison under different levels of electromagnetic interference (site B).

Test period	SNR Range (dB)	Diagnostic accuracy (%)	Positioning error (m)	Main source of interference
High-interference period (07:00–18:00)	25-28	89.7	2.05	The inverter equipment operates with the switch frequently
Low-interference period (02:00–05:00)	35-40	93.1	1.71	The environment is relatively quiet when the variable frequency equipment is shut down.

(4) Excavation Verification Case Studies

Three typical cases are selected for excavation verification.

Case 1 (Site A Corrosion Failure): The model predicted the failure to occur on the third horizontal conductor on the east side, 15.2 meters from the northeast corner grounding electrode. Excavation verification revealed the actual corrosion location at 15.8 meters, with a prediction deviation of 0.6 meters. The corroded section spans approximately 1.2 meters, exhibiting a 40% reduction in cross-sectional area. The conductor surface shows significant rust and pitting. The measured resistance of this section is 0.18 Ω, 2.6 times higher than the normal section (0.05 Ω).

Case 2 (Loose Connection at Site B): The model pinpointed the fault at the southwest corner connection point, predicting coordinates (23.5m, 18.3m). Excavation verification revealed the actual loose connection point at (24.1m, 18.9m), with an Euclidean distance deviation of 0.8 meters. The measured contact resistance at this point was 1.8 Ω, aligning well with the contact resistance range (1.5–2.0 Ω) inferred by the model based on potential distribution anomalies.

Case 3 (Multiple Concurrent Faults at Site A): The model identified two anomalies, located at the north conductor (predicted corrosion) and the east grounding electrode (predicted degradation). Excavation verification confirmed the presence of both faults, with a 1.5-meter deviation at the corrosion location and a 2.3-meter deviation at the degraded grounding electrode. This case demonstrates the model’s capability to identify multiple concurrent faults, but also indicates a reduction in localization accuracy under the influence of multiple interacting faults.

## 5. Conclusions

This article proposed a fault diagnosis and localization method for grounding networks based on graph neural networks and multi-source information fusion (MM-GNN). This method explicitly modeled the physical structure of the grounding grid as a graph, with the physical properties of the conductor segments as edge features. It designed a Physical Perception Topology Graph Convolution (TGGC) based on Ohm’s Law, constructed a sample-adaptive multimodal fusion mechanism, and introduced a topological constraint loss function, thereby achieved a complete solution from modeling, feature extraction, information fusion to end-to-end conductor-level positioning.

The experimental verification results showed that the proposed method achieved a fault diagnosis accuracy of 96.8% on the simulation dataset, reduced the positioning error to 1.23 meters, and took 2.3 seconds for computation. It was significantly better than baseline methods such as CNN-MSF, LSTM-MSF, Trans-MSF, and Std-GNN in both accuracy and efficiency. The on-site test results further indicated that in real complex environments (e.g., multi-layer soil structures and strong electromagnetic interference), the diagnostic accuracy of the proposed method remained at 91.3%, the positioning error was 1.87 meters, and the degree of performance degradation was the smallest among all compared methods, which verified the robustness and reliability of the method in practical engineering environments. The ablation experiment results further validated the effectiveness of each key module and indicated that the synergistic effect of multimodal information fusion and topology-aware graph convolution was an important foundation for the performance improvement.

This study had significant theoretical and practical implications. At the theoretical level, the proposed modeling paradigm (embedding physical attributes into graph edge features), the interpretable attention mechanism based on Ohm’s law, and the topological constraint loss function provided valuable methodological frameworks for the design of graph neural networks for physical networks. The above framework was not limited to the grounding network domain and was theoretically applicable to other power network diagnostic tasks with explicit physical graph structures, such as transmission line fault location and distribution network state perception, thus showed strong method transferability. At the practical level, the proposed method achieved precise fault location at the conductor level, broke through the limitations of traditional regional-level positioning methods, and provided more effective technical support for the safe and stable operation of power systems.

Future work will focus on three directions: ①Model quantification and lightweighting for embedded edge hardware, achieving real-time online diagnosis and incremental adaptive updates with controllable accuracy. ②Introducing domain adaptation and few sample learning, high-quality cross station migration can be achieved with only a small number of annotated samples from the target substation, solving the domain shift problem caused by different soil conditions, grid specifications, and operating years. ③By deeply integrating MM-GNN with the grounding grid digital twin system, the simulation data is continuously used to expand the training samples, and the measured data is used to calibrate the twin model parameters in real time. A closed-loop intelligent diagnostic system that combines simulation and measurement is constructed to enhance the long-term adaptability and engineering credibility of the method.

## Supporting information

S1 Data Data. (XLSX)
